# Synthetic biology open language (SBOL) version 3.0.0

**DOI:** 10.1515/jib-2020-0017

**Published:** 2020-06-25

**Authors:** Hasan Baig, Pedro Fontanarrosa, Vishwesh Kulkarni, James Alastair McLaughlin, Prashant Vaidyanathan, Bryan Bartley, Jacob Beal, Matthew Crowther, Thomas E. Gorochowski, Raik Grünberg, Goksel Misirli, James Scott-Brown, Ernst Oberortner, Anil Wipat, Chris J. Myers

**Affiliations:** University of Connecticut, Storrs, USA; University of Utah, Salt Lake City, USA; University of Warwick, Coventry, UK; Newcastle University, Newcastle, UK; Microsoft Research, Cambridge, UK; Raytheon BBN Technologies, Cambridge, USA; University of Bristol, Bristol, UK; King Abdullah University for Science and Technology, Thuwal, Saudi Arabia; Keele University, Keele, UK; University of Oxford, Oxford, UK; DOE Joint Genome Institute, Walnut Creek, USA

**Keywords:** bioengineering, biological design, data standards, synthetic biology

## Abstract

Synthetic biology builds upon genetics, molecular biology, and metabolic engineering by applying engineering principles to the design of biological systems. When designing a synthetic system, synthetic biologists need to exchange information about multiple types of molecules, the intended behavior of the system, and actual experimental measurements. The Synthetic Biology Open Language (SBOL) has been developed as a standard to support the specification and exchange of biological design information in synthetic biology, following an open community process involving both wet bench scientists and dry scientific modelers and software developers, across academia, industry, and other institutions. This document describes SBOL 3.0.0, which condenses and simplifies previous versions of SBOL based on experiences in deployment across a variety of scientific and industrial settings. In particular, SBOL 3.0.0, (1) separates sequence features from part/sub-part relationships, (2) renames Component Definition/Component to Component/Sub-Component, (3) merges Component and Module classes, (4) ensures consistency between data model and ontology terms, (5) extends the means to define and reference Sub-Components, (6) refines requirements on object URIs, (7) enables graph-based serialization, (8) moves Systems Biology Ontology (SBO) for Component types, (9) makes all sequence associations explicit, (10) makes interfaces explicit, (11) generalizes Sequence Constraints into a general structural Constraint class, and (12) expands the set of allowed constraints.

***Corresponding author: Chris J. Myers**, University of Utah, Salt Lake City, USA, E-mail: myers@ece.utah.edu.
**Hasan Baig:** University of Connecticut, Storrs, USA, E-mail: hasanbaigg@gmail.com. https://orcid.org/0000-0002-8765-2604
**Pedro Fontanarrosa:** University of Utah, Salt Lake City, USA, E-mail: pfontanarrosa@gmail.com. https://orcid.org/0000-0002-0535-2684
**Vishwesh Kulkarni:** University of Warwick, Coventry, UK, E-mail: V.Kulkarni@warwick.ac.uk. https://orcid.org/0000-0002-2285-8652
**James Alastair McLaughlin, Matthew Crowther and Anil Wipat:** Newcastle University, Newcastle, UK, E-mail: james@mclgh.net (J.A. McLaughlin), M.Crowther1@newcastle.ac.uk (M. Crowther), anil.wipat@newcastle.ac.uk (A. Wipat). https://orcid.org/0000-0002-8361-2795 (J.A. McLaughlin) . https://orcid.org/0000-0001-7310-4191 (A. Wipat)
**Prashant Vaidyanathan:** Microsoft Research, Cambridge, UK, E-mail: t-prva@microsoft.com. https://orcid.org/0000-0002-9722-7089
**Bryan Bartley and Jacob Beal:** Raytheon BBN Technologies, Cambridge, USA, E-mail: bartleyba@sbolstandard.org (B. Bartley), jakebeal@gmail.com (J. Beal). https://orcid.org/0000-0002-1597-4022 (B. Bartley) . https://orcid.org/0000-0002-1663-5102 (J. Beal)
**Thomas E. Gorochowski:** University of Bristol, Bristol, UK, E-mail: thomas.gorochowski@bristol.ac.uk. https://orcid.org/0000-0003-1702-786X
**Raik Grünberg:** King Abdullah University for Science and Technology, Thuwal, Saudi Arabia, E-mail: raik.gruenberg@gmail.com. https://orcid.org/0000-0001-9532-6043
**Goksel Misirli:** Keele University, Keele, UK, E-mail: gokselmisirli@gmail.com. https://orcid.org/0000-0002-2454-7188
**James Scott-Brown:** University of Oxford, Oxford, UK, E-mail: james@jamesscottbrown.com. https://orcid.org/0000-0001-5642-8346
**Ernst Oberortner:** DOE Joint Genome Institute, Walnut Creek, USA, E-mail: e.oberortner@gmail.com. https://orcid.org/0000-0002-4287-5513

## Synthetic Biology Open Language (SBOL) Version 3.0

**Editors:**

Hasan Baig — *University of Connecticut, USA*
Pedro Fontanarrosa — *University of Utah, USA*
Vishwesh Kulkarni — *University of Warwick, UK*
James McLaughlin — *Newcastle University, UK*
Prashant Vaidyanathan — *Microsoft Research, UK*

editors@sbolstandard.org

**Chair:**

Chris Myers — *University of Utah, USA*

**Additional authors:**

Bryan Bartley — *Raytheon BBN, USA*
Jacob Beal — *Raytheon BBN Technologies, USA*
Matthew Crowther — *Newcastle University, UK*
Thomas Gorochowski — *University of Bristol, UK*
Raik Grünberg — *KAUST, SA*
Goksel Misirli — *Keele University, UK*
Ernst Oberortner — *DOE Joint Genome Institute, USA*
James Scott-Brown — *University of Oxford, UK*
Anil Wipat — *Newcastle University, UK*

DRAFT

Version 3.0

April 1, 2020

## Contents

1 Purpose 4
2 A Brief History of SBOL 6
3 Overview of SBOL 8
4 Conventions 10
4.1 Terminology Conventions 10
4.2 UML Diagram Conventions 10
4.3 Naming and Typographic Conventions 11
5 Identifiers and Primitive Types 12
5.1 Uniform Resource Identifiers 12
5.2 Primitive Data Types 12
6 SBOL Data Model 14
6.1 Identified 14
6.2 TopLevel 15
6.3 Sequence 15
6.4 Component 17
6.4.1 Feature 21
6.4.1.1 SubComponent 21
6.4.1.2 ComponentReference 23
6.4.1.3 LocalSubComponent 24
6.4.1.4 ExternallyDefined 24
6.4.1.5 SequenceFeature 24
6.4.2 Location 24
6.4.2.1 Range 25
6.4.2.2 Cut 25
6.4.2.3 EntireSequence 26
6.4.3 Constraint 26
6.4.4 Interaction 27
6.4.4.1 Participation 29
6.4.5 Interface 30
6.5 CombinatorialDerivation 31
6.5.1 VariableComponent 32
6.6 Implementation 34
6.7 ExperimentalData 34
6.8 Model 35
6.9 Collection 36
6.9.1 Namespace 37
6.9.2 Experiment 37
6.10 Attachment 37
6.11 Annotation and Extension of SBOL 38
7 Recommended Best Practices 39
7.1 SBOL Versions 39
7.2 Compliant SBOL Objects 39
7.3 Versioning SBOL Objects 40
7.4 Annotations: Embedded Objects vs. External References 40
7.5 Completeness and Validation 40
7.6 Recommended Ontologies for External Terms 40
7.7 Annotating Entities with Date & Time 41
7.8 Annotating Entities with Authorship information 41
7.9 Host Context / Ontologies for Experiments 41
7.9.1 Mixtures via Components 41
7.9.2 Media, Inducers, and Other Reagents 41
7.9.3 Samples 42
7.9.4 Other Experimental Parameters 42
7.10 Multicellular System Designs 42
7.10.1 Representing Cell Types 43
7.10.2 Multiple Cell Types in a Single Design 43
7.10.3 Cell Ratios 43
8 SBOL RDF Serialization 46
9 SBOL Compliance 47

**10 Mapping Between SBOL 1, SBOL 2, and SBOL3** **48**
10.1 Mapping between SBOL 1 and SBOL 2 . . . . . 48
10.2 Mapping between SBOL 2 and SBOL 3 . . . . . 48
**References** **52**
**A Complementary Standards** **53**
A.1 Adding Provenance with PROV-O . . . . . 53
A.1.1 prov:Activity . . . . . 54
A.1.2 prov:Usage . . . . . 56
A.1.3 prov:Association . . . . . 56
A.1.4 prov:Plan . . . . . 56
A.1.5 prov:Agent . . . . . 56
A.2 Adding Measures/Parameters with OM . . . . . 58
A.2.1 om:Measure . . . . . 58
A.2.2 om:Unit . . . . . 59
A.2.3 om:SingularUnit . . . . . 60
A.2.4 om:CompoundUnit . . . . . 60
A.2.5 om:UnitMultiplication . . . . . 60
A.2.6 om:UnitDivision . . . . . 60
A.2.7 om:UnitExponentiation . . . . . 61
A.2.8 om:PrefixedUnit . . . . . 61
A.2.9 om:Prefix . . . . . 61
A.2.10 om:SIPrefix . . . . . 62
A.2.11 om:BinaryPrefix . . . . . 62

## 1 Purpose

Synthetic biology builds upon genetics, molecular biology, and metabolic engineering by applying engineering principles to the design of biological systems. When designing a synthetic system, synthetic biologists need to exchange information about multiple types of molecules, the intended behavior of the system, and actual experimental measurements. Furthermore, there are often multiple aspects to a design such as a specified nucleic acid sequence (e.g., a sequence that encodes an enzyme or transcription factor), the molecular interactions that a designer intends to result from the introduction of this sequence (e.g., chemical modification of metabolites or regulation of gene expression), and the experiments and data associated with the system. All these perspectives need to be connected together to facilitate the engineering of biological systems.

The *Synthetic Biology Open Language* (SBOL) has been developed as a standard to support the specification and exchange of biological design information in synthetic biology, following an open community process involving both "wet" bench scientists and "dry" scientific modelers and software developers, across academia, industry, and other institutions. Previous nucleic acid sequence description formats lack key capabilities relative to SBOL, as shown in Figure 1. Simple sequence encoding formats such as FASTA encode little besides sequence information. More sophisticated formats such as GenBank and Swiss-Prot provide a flat annotation of sequence features that is well suited to describing natural systems but unable to represent the functional relations and multi-layered design structure common to engineered systems. Modeling languages, such as the Systems Biology Markup Language (SBML) Hucka et al. (2003), can be used represent biological processes, but are not sufficient to represent the associated nucleotide or amino acid sequences. SBOL covers both of these needs, by providing a modular and hierarchical representation of the structure and function of a genetic design, as well as its relationship to and use within experiment plans, data, models, etc.

SBOL uses existing Semantic Web practices and resources, such as *Uniform Resource Identifiers* (`URI`s) and ontologies, to unambiguously identify and define biological system elements, and to provide serialization formats for encoding this information in electronic data files. The SBOL standard further describes the rules and best practices on how to use this data model and populate it with relevant design details. The definition of the data model, the rules on the addition of data within the format, and the representation of this in electronic data files are intended to make the SBOL standard a useful means of promoting data exchange between laboratories and between software programs.

### Differences from Prior Versions of SBOL

SBOL 1 focused on representing the structural aspects of genetic designs: it allowed the exchange of information about DNA designs and their sequence features, but could not represent molecules other than DNA or the functional aspects of designs. SBOL 2 enabled the description and exchange of hierarchical, modular representations of both the intended structure and function of designed biological systems, as well as providing support for representing provenance, combinatorial designs, genetic design implementations, external file attachments, experimental data, and numerical measurements. SBOL 3.0, defined by this document, condenses and simplifies these prior representations based on experiences in deployment across a variety of scientific and industrial settings.

Specifically, SBOL 3.0 improves on its predecessor SBOL 2.3 by:

- Separating sequence features from part/sub-part relationships.
- Renaming ComponentDefinition/Component to Component/SubComponent.
- Merging Component and Module classes.
- Ensuring consistency between data model and ontology terms.
- Extending the means to define and reference SubComponents.
- Refining requirements on object URIs.

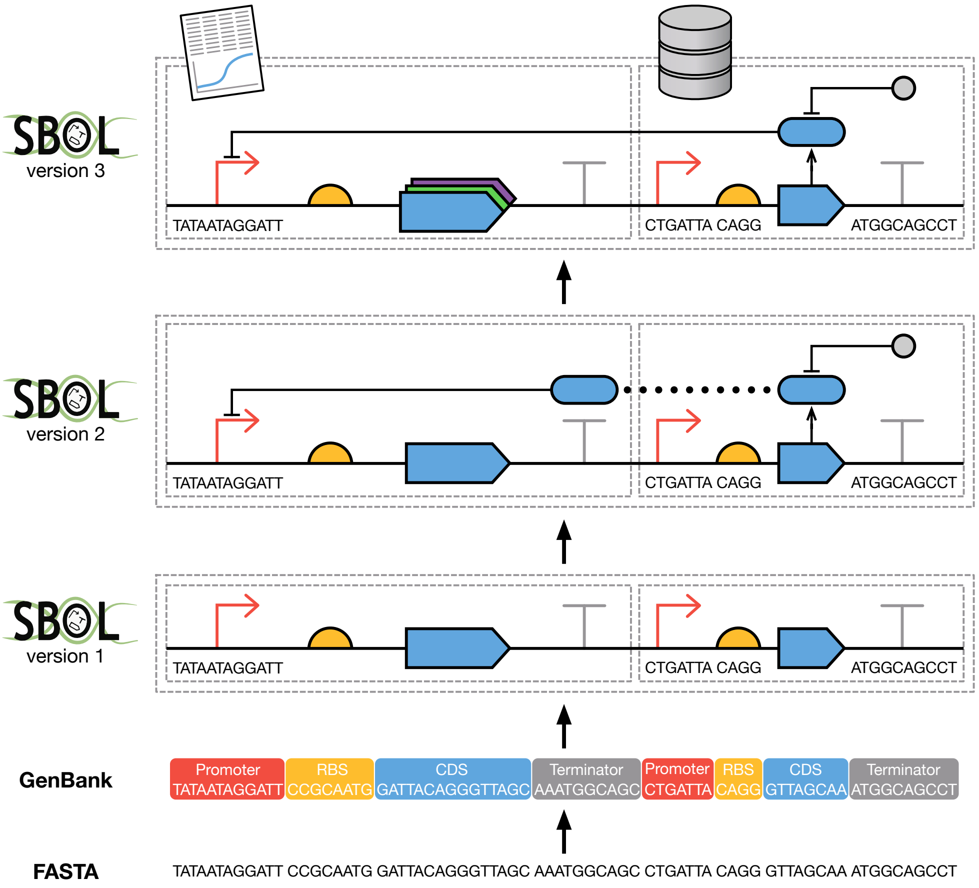

Figure 1: SBOL extends prior sequence description formats to represent both the structure and function of a genetic design in a modular, hierarchical manner, as well as its relationship to, and use within, experiments, plans, data, models, etc.

- Enabling graph-based serialization.
- Moving to Systems Biology Ontology (SBO) for Component types.
- Making all sequence associations explicit.
- Making interfaces explicit.
- Generalizing SequenceConstraints into a general structural Constraint class.
- Expanding the set of allowed sequence constraints.

## 2 A Brief History of SBOL

The SBOL effort was started in 2006 with the goal of developing a data exchange standard for genetic designs. Herbert Sauro (University of Washington) secured a grant from Microsoft in the field of computational synthetic biology, which was used to fund the initial meeting in Seattle on April 26-27, 2008. This workshop was organized by Herbert Sauro, Sean Sleight, and Deepak Chandran, and included talks by Raik Gruenberg, Kim de Mora, John Cumbers, Christopher Anderson, Mac Cowell, Jason Morrison, Jean Peccoud, Ralph Santos, Andrew Milar, Vincent Rouilly, Mike Hucka, Michael Blinov, Lucian Smith, Sarah Richardson, Guillermo Rodrigo, Jonathan Goler, and Michal Galdzicki.

Michal's early efforts were instrumental in making SBOL successful. As part of his doctoral work, he led the development of PoBol (Provisional BioBrick Language), as SBOL was originally known. He organized annual workshops from 2008 to 2011 and kept the idea of developing a genetic design standard alive. The original SBOL 1.0 was developed by a small group of dedicated researchers calling themselves the Synthetic Biology Data Exchange Working Group, meeting at Stanford in 2009 and Anaheim, CA in 2010. During the Anaheim meeting, the community decided to write a letter to Nature Biotechnology highlighting the issue of reproducibility in synthetic biology Peccoud et al. (2011). This letter was initiated by Jean Peccoud and submitted by participants of the Anaheim meeting, including Deepak Chandran, Douglas Densmore, Dmytriv, Michal Galdzicki, Timothy Ham, Cesar Rodriquez, Jean Peccoud, Herbert Sauro, and Guy-Bart Stan. The overall pace of development quickened when several new members joined at the next workshop in Blacksburg, Virginia on January 7-10, 2011. This early work was also supported by an STTR grant from the National Institute of Health (NIH #1R41LM010745 and #9R42HG006737, from 2010-13) in collaboration with Clark & Parsia, LLC (Co-PIs: John Gennari and Evren Sirin). New members included Cesar Rodriguez, Mandy Wilson, Guy-Bart Stan, Chris Myers, and Nicholas Roehner.

The SBOL Developers Group was officially established at a meeting in San Diego in June 2011. Rules of governance were established, and the first SBOL editors were elected: Mike Galdzicki, Cesar Rodriguez, and Mandy Wilson. At our next meeting in Seattle in January 2012, Herbert Sauro was elected the SBOL chair, and two new editors were added: Matthew Pocock and Ernst Oberortner. New developers joining at these workshops included several representatives from industry, Kevin Clancy, Jacob Beal, Aaron Adler, and Fusun Yaman Sirin. New members from Newcastle University included Anil Wipat, Matthew Pocock, and Goksel Misirli.

Development of the first software library (libSBOLj) based on the SBOL standard was initiated by Allan Kuchinsky, a research scientist from Agilent, at the 2011 meeting. By the time of the 2012 meeting, the first data exchange between software tools using SBOL was conducted when a design was passed from Newcastle University's VirtualParts Repository to Boston University's Eugene tool, and finally to University of Utah's iBioSim tool.

SBOL 1.0 was officially released in October 2011. In March 2012, SBOL 1.1 was released, the version that this document replaces. SBOL 1.1 did not make any major changes, but provided a number of small adjustments and clarifications, particularly around the annotation of sequences. Multi-institutional data exchange using SBOL 1.1 was later demonstrated in Nature Biotechnology Galdzicki et al. (2014).

While SBOL 1.1 had a number of significant advantages over the GenBank representation of DNA sequences, such as representing hierarchical organization of DNA components, it was still limited in other respects. The major topic of discussion at the 8th SBOL Workshop at Boston University in November 2012 was how to address these shortcomings through extensions. Several extensions were discussed at this meeting, such as a means to describe genetic regulation, which later became important classes in the current 2.x specification.

A general framework for SBOL 2.0 emerged at the 9th SBOL workshop at Newcastle University in April 2013. Subsequently, Nicholas Roehner, Matthew Pocock, and Ernst Oberortner drafted a proposal for SBOL 2.0, and Nicholas presented this proposal at the SEED conference in Los Angeles in July 2014 Roehner et al. (2015). The proposed 2.0 data model was discussed over the course of the 10th, 11th, and 12th workshops. The SBOL 2.0 specification document was drafted at the 13th workshop in Wittenberg, Germany. The SBOL 2.x data model presented was essentially the result of these meetings and ongoing discussions conducted through the SBOL Developers mailing lists, plus minor adjustments and updates approved by the community through subsequence meetings and mailing list discussions.

From 2014 to 2019, development of SBOL 2.x was funded in large part by a grant from the National Science Foundation (DBI-1355909 and DBI-1356041). The SBOL 2.x specification documents and the supporting software libraries are due in no small part to this support. Any opinions, findings, and conclusions or recommendations expressed in SBOL materials are those of the author(s) and do not necessarily reflect the views of the National Science Foundation.

The Computational Modeling in Biology Network (COMBINE) holds regular workshops at which synthetic biologists and systems biologists work toward a common goal of integrating biological knowledge through interoperable and non-overlapping data standards. Several SBOL Developers proposed that SBOL join this larger standards community after attended a COMBINE workshop in April 2014. The proposal passed and SBOL workshops have been co-located with COMBINE meetings since the 11th workshop at the University of Southern California in August 2014.

In 2019 the SBOL Industrial Consortium was established as a pre-competitive non-profit organization supporting innovation, dissemination, and integration of SBOL standards, tools and practices for practical applications in an industrial environment. The SBOL Industrial Consortium meets regularly to coordinate its activities, and organises an Industrial Advisory Board to give an industrial perspective on SBOL, as well as providing financial support for projects, activities, and infrastructure within the SBOL community. Member organsiations include Raytheon BBN Technologies, Doulix, Integrated DNA Technologies, Twist Bioscience, Amyris, Inscripta, Teselagen, Shipyard Toolchains, and Zymergen.

Discussions related to SBOL 3 began at the COMBINE meetings and on the mailing list beginning in the summer of 2018. Over the next year and a half, several SBOL Enhancement Proposals (SEPs) were written and discussed. During the early months of 2020, these SEPs were voted on and approved by the SBOL community. The initial version of the SBOL 3 specification was drafted during HARMONY 2020 at the European Bioinformatics Institute (EBI) in Hinxton, United Kingdom in March 2020.

The authors would also like to thank Michael Hucka for developing the LaTeX style file used to develop this document (Hucka, 2017).

## 3 Overview of SBOL

Synthetic biology designs can be described using:

- Structural terms, e.g., a set of annotated sequences or information about the chemical makeup of components.
- Functional terms, e.g., the way that components might interact with each other.

As an example, consider an expression cassette, such as the one found in the plasmid pUC18 Norrander et al. (1983). The system is designed to visually indicate whether a gene has been inserted into the plasmid: in the presence of IPTG, it expresses an enzyme that hydrolyses X-gal to form a blue product, but successful insertion disrupts the expression cassette and prevents the formation of this product. Internally, it has a number of parts, including a promoter, the lac repressor binding site, and the lacZ coding sequence. These parts have specific component-level interactions with IPTG and X-gal, as well as native host gene products, transcriptional machinery, and translational machinery that collectively cause the desired system-level behavior.

In SBOL 3, both the structural and functional aspects are described using a class called `Component`, as depicted in Figure 2. Namely, to represent structural aspects, a `Component` can include `Feature`s, some of which may be at some `Location` within a `Sequence`. A `Component` can also include `Constraint`s between these features. To represent functional aspects, a `Component` can include `Interaction`s that can refer to relationships between participating `Feature`s. Finally, a `Component` can have its behavior described using a `Model`.

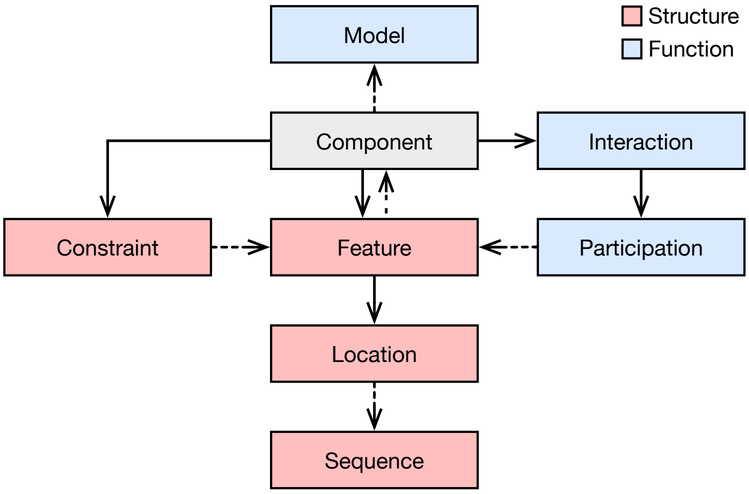

Figure 2: The SBOL `Component` object and related objects. Solid arrows indicates ownership, whereas a dashed arrow represents a reference to an object of another class. Red boxes represent structural objects, while blue boxes represent functional objects. To represent structural aspects, a `Component` can include `Feature`s, which may refer to `Location`s within a `Sequence`. A `Component` can also include `Constraint`s between these features. To represent functional aspects, a `Component` can include `Interaction`s that can refer to relationships between participating `Feature`s. Finally, a `Component` can have its behavior described using a `Model`.

To continue with the pUC18 example, the description would begin with a top-level `Component` that represents the entire system. This `Component` specifies the structural elements that make up the cassette by referencing a number of `SubComponent` objects. These would include the DNA `SubComponent` for the promoter and the simple chemical `SubComponent` for IPTG, for example. The `Component` objects can be organized hierarchically. For example, the plasmid `Component` might reference `SubComponent`s for the promoter, coding sequence, etc. Each `Component` object can also include the actual Sequence information (if available), as well as SubComponent objects that identify the Locations of the promoters, coding sequences, etc., on the Sequence. In order to specify functional information, the Component can also specify Interaction objects that describe any qualitative relationships among SubComponent Participations, such as how IPTG and X-gal interact with the gene products. Finally, a Component object can point to a Model object that provides a reference to a complete computational model expressed in a language such as SBML Hucka et al. (2003), CellML Cuellar et al. (2003), or MATLAB MathWorks (2015).

Whereas Figure 2 provides an overview of the classes used for describing designs within the SBOL 3 data model, Figure 3 shows the rest of the classes used to describe the usage of a design within design-build-test-learn workflows in general. In particular, designs can be expressed using CombinatorialDerivations, Components, and Sequences. These can describe not only genetic designs, but also designs for strains, multicellular systems, media, samples, etc. A CombinatorialDerivation allows one to specify a design pattern where individual SubComponents can be selected from a set of variants. The Implementation class is the build class, and it is used to represent physical artifacts like an actual sample of a plasmid. The Experiment and ExperimentalData classes are the test classes, allowing description of a collection of data generated in an experiment. The Model class, discussed earlier, associates learned information with a design. The prov:Activity class is taken from the provenance ontology (PROV-O), which is described in Section A.1. For example, a build prov:Activity describes how an Implementation is constructed using a Component description. On the other hand, a test prov:Activity describes how an Experiment is conducted using an Implementation artifact. The Collection class has members, which can be of any of these types or Collections themselves. Finally, all of these objects can refer to objects of the Attachment class, which are used to link out to external data (images, spreadsheets, textual documents, etc.). The next sections provide complete definitions and details for all of these classes.

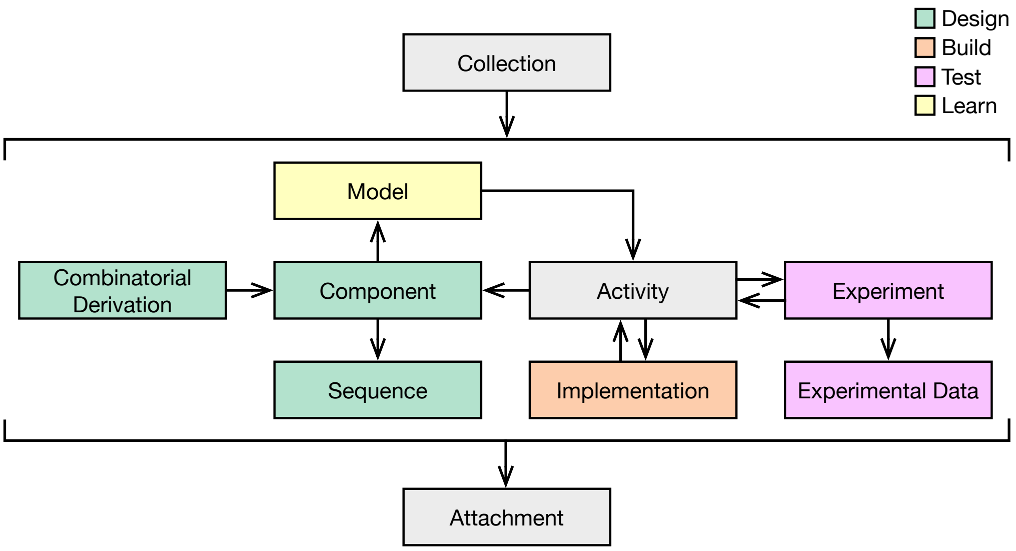

Figure 3: Main classes of information represented by the SBOL 3 standard, and their relationships. Green boxes represent design classes, orange boxes represent build classes, purple boxes represent test classes, yellow boxes represent learn classes, and the gray boxes represent additional utility classes. Each of these classes will be described in more detail below.

## 4 Conventions

This section provides some preliminary information to aid in the understanding of the specification. The SBOL data model is specified using Unified Modeling Language (UML) 2.0 diagrams (OMG 2005). This section reviews terminology conventions, the basics of UML diagrams, and our naming conventions.

### 4.1 Terminology Conventions

This document indicates requirement levels using the controlled vocabulary specified in IETF RFC 2119. In particular, the key words "MUST", "MUST NOT", "REQUIRED", "SHALL", "SHALL NOT", "SHOULD", "SHOULD NOT", "RECOMMENDED", "MAY", and "OPTIONAL" in this document are to be interpreted as described in RFC 2119.

- The words "MUST", "REQUIRED", or "SHALL" mean that the item is an absolute requirement.
- The phrases "MUST NOT" or "SHALL NOT" mean that the item is an absolute prohibition.
- The word "SHOULD" or the adjective "RECOMMENDED" mean that there might exist valid reasons in particular circumstances to ignore a particular item, but the full implications need to be understood and carefully weighed before choosing a different course.
- The phrases "SHOULD NOT" or "NOT RECOMMENDED" mean that there might exist valid reasons in particular circumstances when the particular behavior is acceptable or even useful, but the full implications needs to be understood and the case carefully weighed before implementing any behavior described with this label.
- The word "MAY" or the adjective "OPTIONAL" mean that an item is truly optional.

### 4.2 UML Diagram Conventions

The types of biological design data modeled by SBOL are commonly referred to as *classes*, especially when discussing the details of software implementation. Each SBOL class can be instantiated by many SBOL objects. These objects MAY contain data that differ in content, but they MUST agree on the type and form of their data as dictated by their common class. Classes are represented in UML diagrams as rectangles labeled at the top with class names (see Figure 4 for examples).

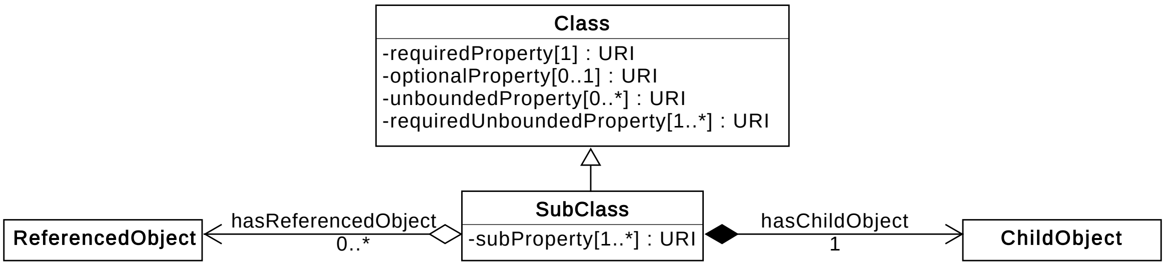

Figure 4: Examples of UML diagram conventions used in this document

Classes can be connected to other classes by association properties, which are represented in UML diagrams as arrows. These arrows are labeled with data cardinalities in order to indicate how many values a given association property can possess (see below). The remaining (non-association) properties of a class are listed below its name. Each of the latter properties is labeled with its data type and cardinality.

In the case of an association property, the class from which the arrow originates is the owner of the association property. A diamond at the origin of the arrow indicates the type of association. Open-faced diamonds indicate shared aggregation, also known as a reference, in which the owner of the association property exists independently of its value.

By contrast, filled diamonds indicate composite aggregation, also known as a part-whole relationship, in which the value of the association property MUST NOT exist independently of its owner. In addition, in the SBOL data model, it is REQUIRED that the value of each composite aggregation property is a unique SBOL object (that is, not the value for more than one such property). Note that in all cases, composite aggregation is used in such a way that there SHOULD NOT be duplication of such objects. Such objects are also commonly referred to as "child" objects, and their owning objects as "parent" objects.

All SBOL properties are labeled with one of several restrictions on data cardinality. These are:

- 1 - REQUIRED, one: there MUST be exactly one value for this property.
- $0 \ldots 1$ - OPTIONAL: there MAY be a single value for this property, or it MAY be absent.
- $0 \ldots *$ - unbounded: there MAY be any number of values for this property, including none.
- $1 \ldots *$ - REQUIRED, unbounded: there MAY be any number of values for this property, as long as there is at least one.
- $n \ldots *$ - at least: there MUST be at least $n$ values for this property.

Finally, classes can inherit the properties of other classes. Inheritance relationships are represented in UML diagrams as open-faced, triangular arrows that point from the inheriting class to the inherited class. Some classes in the SBOL data model cannot be instantiated as objects and exist only to group common properties for inheritance. These classes have italicized names and are known as abstract classes.

### 4.3 Naming and Typographic Conventions

SBOL classes are named using upper "camel case," meaning that each word is capitalized and all words are run together without spaces, e.g., `Identified`, `SequenceFeature`. Properties, on the other hand, are named using lower camel case, meaning that they begin lowercase (e.g., `role`) but if they consist of multiple words, all words after the first begin with an uppercase letter (e.g., `roleIntegration`). SBOL properties are always given singular names irrespective of their cardinality, e.g., `role` is used rather than `role` even though a component can have multiple roles. This is because each relation can potentially stand on its own, irrespective of the existence of others in the set.

## 5 Identifiers and Primitive Types

### 5.1 Uniform Resource Identifiers

As SBOL is built upon the Resource Description Framework (RDF), all class instances are identified by a Uniform Resource Identifier (URI). In the SBOL data model, the value of an association property MUST be a `URI` or set of `URI`s that refer to SBOL objects belonging to the class at the tip of the arrow. Every `Identified` object's URI MUST be globally unique among all other `Identified` object URIs. It is also highly RECOMMENDED that the `URI` structure follows the recommended best practices for compliant `URI`s specified in Section 7.2.

Whenever a `TopLevel` object's URI is a URL (e.g., following the conventions of HTTP(S) rather than a UUID), its structure MUST comply with the following rules:

- A `TopLevel` URL MUST use the following pattern: `[namespace]/[local]/[displayId]`, where `namespace` and `displayId` are required fragments, and the `local` fragment is an optional relative path. For example, a `Component` might have the URL `https://synbiohub.org/public/igem/BBa_J23070`, where `namespace` is `https://synbiohub.org`, `local` is `public/igem`, and `displayId` is `BBa_J23070`.

- A `TopLevel` object's URL MUST NOT be included as prefix for any other `TopLevel` object (except for its controlling `Namespace`). For example, the `BBa_J23070_seq` `Sequence` object cannot have a URL of `https://synbiohub.org/public/igem/BBa_J23070/BBa_J23070_seq`, since the `https://synbiohub.org/public/igem/BBa_J23070` prefix is already used as a URL for the `BBa_J23070` `Component` object.

- The URL of any child or nested object MUST use the following pattern: `[parent]/[displayId]`, where `parent` is the URL of its parent object. Multiple layers of child objects are allowed using the same `[parent]/[displayId]` pattern recursively. For example, a `SequenceFeature` object owned by the `BBa_J23070` `Component` and having a `displayId` of `annotation1` will have a URL of `https://synbiohub.org/public/igem/BBa_J23070/annotation1`. Similarly, the `loc1` `Location` child of the `annotation1` `SequenceFeature` object will have the URL `https://synbiohub.org/public/igem/BBa_J23070/annotation1/loc1`.

### 5.2 Primitive Data Types

When SBOL uses simple "primitive" data types such as `String`s or `Integer`s, these are defined as the following specific formal types:

- `String`: `http://www.w3.org/TR/xmlschema11-2/#string`
  *Example: "LacI coding sequence"*

- `Integer`: `http://www.w3.org/TR/xmlschema11-2/#integer`
  *Example: 3*

- `Long`: `http://www.w3.org/TR/xmlschema11-2/#long`
  *Example: 9223372036854775806*

- `Double`: `http://www.w3.org/TR/xmlschema11-2/#double`
  *Example: 3.14159*

- `Boolean`: `http://www.w3.org/TR/xmlschema11-2/#boolean`
  *Example:* `true`

The term `literal` is used to denote an object that can be any of the five types listed above.

In addition to the simple types listed above, SBOL also uses objects with types *Uniform Resource Identifier* (`URI`). It is important to realize that in RDF, a `URI` might or might not be a resolvable URL (web address). A `URI` is always a globally unique identifier within a structured namespace. In some cases, that name is also a reference to (or within) a document, and in some cases that document can also be retrieved (e.g., using a web browser).

## 6 SBOL Data Model

The section describes the SBOL data model in detail. Best practices when using the standard can be found in Section 7.

### 6.1 Identified

All SBOL-defined classes are directly or indirectly derived from the `Identified` abstract class. This inheritance means that all SBOL objects are uniquely identified using `URI`s that uniquely refer to these objects within an SBOL document or at locations on the World Wide Web.

As shown in Figure 5, the `Identified` class includes the following properties: `displayId`, `name`, `description`, `prov:wasDerivedFrom`, and `prov:wasGeneratedBy`.

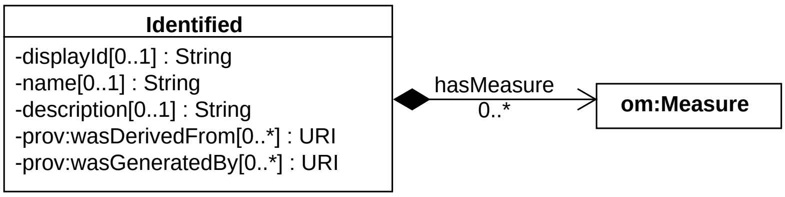

Figure 5: Diagram of the `Identified` abstract class and its associated properties

##### *The `displayId` property*

The `displayId` property is an OPTIONAL identifier with a data type of `String`. This property is intended to be an intermediate between a URI and the `name` property that is machine-readable, but more human-readable than the full URI of an object.

If the `displayId` property is used, then its `String` value MUST be composed of only alphanumeric or underscore characters and MUST NOT begin with a digit.

Note that for objects whose URI is a URL, the requirements on URL structure in Section 5.1 imply that the `displayId` MUST be set.

##### *The `name` property*

The `name` property is OPTIONAL and has a data type of `String`. This property is intended to be displayed to a human when visualizing an `Identified` object.

If an `Identified` object lacks a name, then software tools SHOULD instead display the object's `displayId` or URI. It is RECOMMENDED that software tools give users the ability to switch perspectives between `name` properties that are human-readable and `displayId` properties that are less human-readable, but are more likely to be unique.

##### *The `description` property*

The `description` property is OPTIONAL and has a data type of `String`. This property is intended to contain a more thorough text description of an `Identified` object.

##### *The `prov:wasDerivedFrom` property*

An `Identified` object can have zero or more `prov:wasDerivedFrom` properties, each of type URI. This property is defined by the PROV-O ontology and is located in the `https://www.w3.org/TR/prov-o/` namespace (Reference:

Section A.1).

An SBOL object with this property refers to one or more SBOL objects or non-SBOL resources from which this object was derived. An SBOL object MUST NOT refer to itself via its own prov:wasDerivedFrom property or form a cyclical chain of references via its prov:wasDerivedFrom property and those of other SBOL objects. For example, the reference chain "*A* was derived from *B* and *B* was derived from *A*" is cyclical.

##### *The* prov:wasGeneratedBy *property*

An Identified object can have zero or more prov:wasGeneratedBy properties, each of type URI. This property is defined by the PROV-O ontology and is located in the https://www.w3.org/TR/prov-o/ namespace (Reference: Section A.1).

An SBOL object with this property refers to one or more prov:Activity objects that describe how this object was generated. Provenance history formed by prov:wasGeneratedBy properties of Identified objects and entity references in prov:Usage objects MUST NOT form circular reference chains.

##### *The* hasMeasure *property*

An Identified object can have zero or more hasMeasure properties, each of type URI. This property is defined by the OM ontology and is located in the http://www.ontology-of-units-of-measure.org/resource/om-2/ namespace (Reference: Section A.2).

An SBOL object with this property refers to one or more om:Measure objects that describe measured parameters for this object.

### 6.2 TopLevel

TopLevel is an abstract class that is extended by any Identified class that can be found at the top level of an SBOL document or file. In other words, TopLevel objects are not nested inside any other object via *composite aggregation* (represented by a filled diamond arrowhead on the UML diagrams). Instead of nesting, composite TopLevel objects refer to subordinate TopLevel objects by their URIs using *shared aggregation* (represented by an open-faced/non-filled diamond arrowhead on the UML diagrams). The TopLevel classes defined in this specification are Sequence, Component, Model, Collection, CombinatorialDerivation, Implementation, Attachment, ExperimentalData, prov:Activity, prov:Agent, prov:Plan (see Figure 6). Each of these classes is described in more detail below, except for the classes from the provenance ontology (PROV-O), which are described in Section A.1.

##### *The hasAttachment property*

A TopLevel object can have zero or more hasAttachment properties, each of type URI specifying an Attachment object. The Attachment class is described in more detail in Section 6.10.

### 6.3 Sequence

The purpose of the Sequence class is to represent the primary structure of a Component object and the manner in which it is encoded. This representation is accomplished by means of the elements property and encoding property (Figure 7).

##### *The* elements *property*

The elements property is an OPTIONAL String of characters that represents the constituents of a biological or chemical molecule. For example, these characters could represent the nucleotide bases of a molecule of DNA, the amino acid residues of a protein, or the atoms and chemical bonds of a small molecule.

If the elements property is not set, then it means the particulars of this Sequence have not yet been determined.

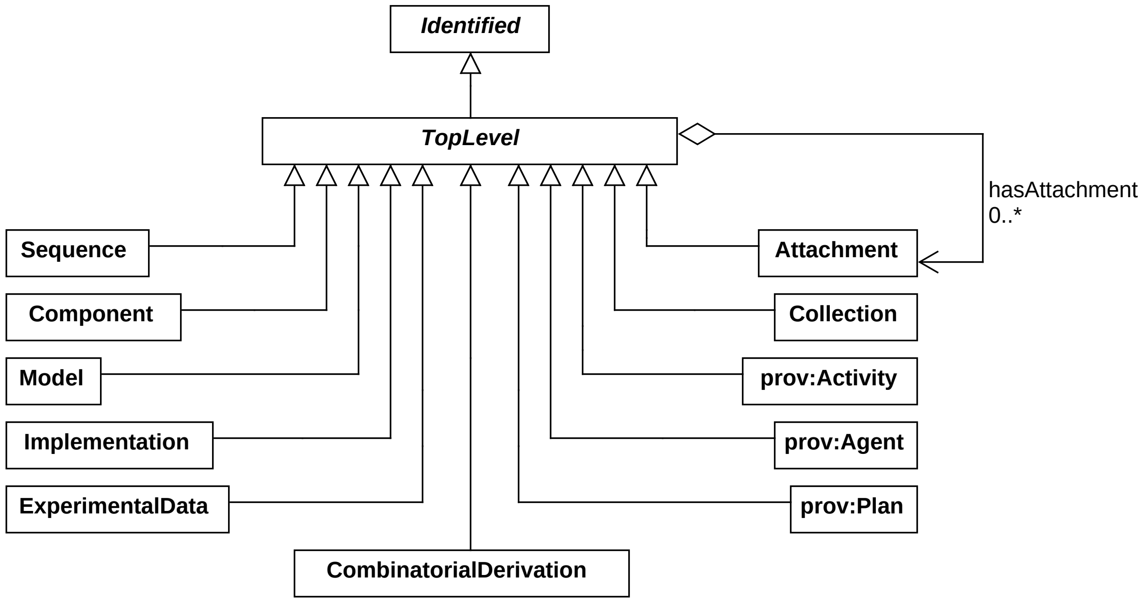

Figure 6: Classes that inherit from the `TopLevel` abstract class.

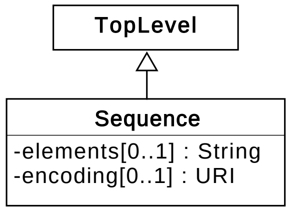

Figure 7: Diagram of the `Sequence` class and its associated properties.

##### *The `encoding` property*

The `encoding` property has a data type of `URI`, and is OPTIONAL unless `elements` is set, in which case it is REQUIRED. This property MUST indicate how the `elements` property of a `Sequence` are formed and interpreted.

For example, the `elements` property of a `Sequence` with an IUPAC DNA encoding property MUST contain characters that represent nucleotide bases, such as `a`, `t`, `c`, and `g`. The `elements` property of a `Sequence` with a Simplified Molecular-Input Line-Entry System (SMILES) encoding, on the other hand, MUST contain characters that represent atoms and chemical bonds, such as `C`, `N`, `O`, and `=`.

Table 1 provides a list of possible `URI` values for the `encoding` property. The terms in Table 1 are organized by the type of `Component` (see Table 2) that typically refer to a `Sequence` with such an `encoding`. It is RECOMMENDED that the encoding property of a Sequence contains a URI from Table 1. When the `encoding` of a `Sequence` is well described by one of the `URI`s in Table 1, it MUST contain that `URI`.

More information on IUPAC encoding can be found at `http://www.bioinformatics.org/sms2/iupac.html`.

| Encoding | URI | Component Type |
|---|---|---|
| IUPAC DNA, RNA | http://sbols.org/v3#iupacNucleicAcid | DNA, RNA |
| IUPAC Protein | http://sbols.org/v3#iupacAminoAcid | Protein |
| SMILES | http://www.opensmiles.org/opensmiles.html | Simple Chemical |

Table 1: URIs for specifying the encoding property of a Sequence, organized by the type of Component (see Table 2) that typically refer to a Sequence with such an encoding.

### 6.4 Component

The Component class represents the structural and/or functional entities of a biological design. The primary usage of this class is to represent entities with designed sequences, such as DNA, RNA, and proteins, but it can also be used to represent any other entity that is part of a design, such as simple chemicals, molecular complexes, strains, media, light, and abstract functional groupings of other entities.

As shown in Figure 8, the Component class describes a design entity using the following properties: type, role, hasSequence, hasFeature, hasConstraint, hasInteraction, hasInterface, and hasModel. The hasSequence, hasFeature, and hasConstraint properties are used to represent structural information, while the hasInteraction, hasInterface, and hasModel are used to represent functional information.

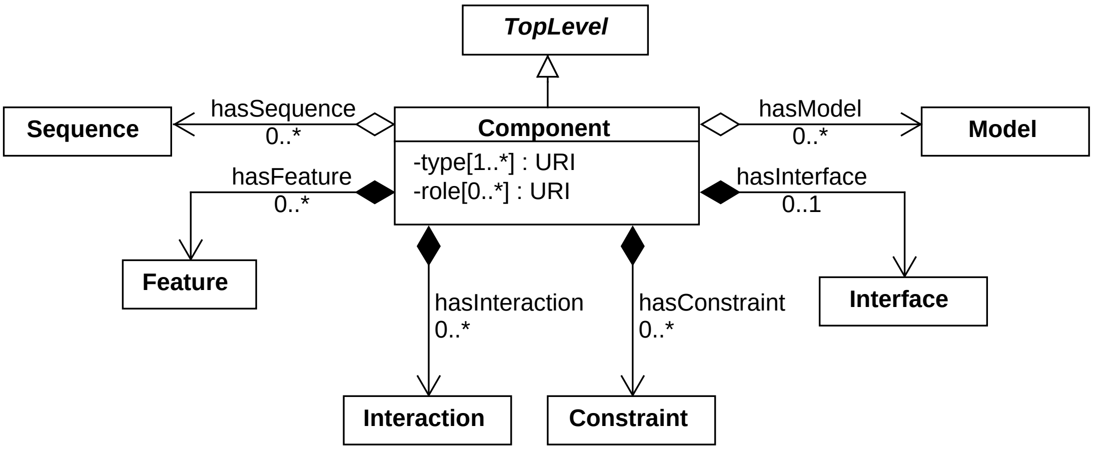

Figure 8: Diagram of the Component class and its associated properties.

##### *The type property*

A Component is REQUIRED to have one or more type properties, each of type URI specifying the category of biochemical or physical entity (for example DNA, protein, or simple chemical) that a Component object abstracts for the purpose of engineering design. For DNA or RNA entities, additional type properties MAY be used to describe nucleic acid topology (circular / linear) and strandedness (double- or single-stranded).

The type properties of every Component MUST include one or more URIs that MUST identify terms from appropriate ontologies, such as the physical entity representation branch of the Systems Biology Ontology Courtot et al. (2011) or the ontology of Chemical Entities of Biological Interest (ChEBI) Degtyarenko et al. (2008). In order to maximize the compatibility of designs, the type property of a Component SHOULD contain a URI from the physical entity representation branch of the Systems Biology Ontology Courtot et al. (2011). Table 2 provides a partial list of ontology terms and their URIs, and any Component that can be well-described by one of the terms in Table 2 MUST

use the URI for that term as a type. Finally, if the type property contains multiple URIs, then they MUST identify non-conflicting terms (otherwise, it might not be clear how to interpret them). For example, the SBO terms provided by Table 2 would conflict because they specify classes of biochemical entities with different molecular structures.

| Component Type | URI for SBO Term |
|---|---|
| DNA (Deoxyribonucleic acid) | https://identifiers.org/SBO:0000251 |
| RNA (Ribonucleic acid) | https://identifiers.org/SBO:0000250 |
| Protein (Polypeptide chain) | https://identifiers.org/SBO:0000252 |
| Simple Chemical | https://identifiers.org/SBO:0000247 |
| Non-covalent complex | https://identifiers.org/SBO:0000253 |
| Functional Entity | https://identifiers.org/SBO:0000241 |

Table 2: Partial list of the most common SBO terms to specify the molecule type using the type property of a Component. Systems of multiple interacting molecules (e.g., a plasmid expressing a protein) should use the functional entity type.

*Nucleic Acid Topology types*

Any Component classified as DNA (see Table 2) is RECOMMENDED to encode circular/linear topology information in an additional type field. This (topology) type field SHOULD specify a URI from the Topology Attribute branch of the Sequence Ontology (SO): this is currently just 'linear' or 'circular' as given in Table 3. Topology information SHOULD be specified for DNA Component records with a fully specified sequence, except in three scenarios: if the DNA record does not have sequence information, or if the DNA record has incomplete sequence information, or if topology is genuinely unknown. For any Component classified as RNA (see Table 2), a topology type field is OPTIONAL. The default assumption in this case is linear topology. In any case, conflicting topologies MUST NOT be specified.

Any Component classified as DNA or RNA MAY also have strand information encoded in an additional (third) type field using a URI from the Strand Attribute branch of the SO (currently there are only two possible terms for single or double-stranded nucleic acids, given in Table 3). In absence of this field, the default strand information assumed for DNA is 'double-stranded' and for RNA is 'single-stranded'.

Any other type of Component record (protein, simple chemical, etc.) SHOULD NOT have any type field pointing to SO terms from the topology or strand attribute branches of SO.

Note that a *circular* topology instructs software to interpret the beginning / end position of a given sequence (be it DNA or RNA) as arbitrary, meaning that sequence features MAY be mapped or identified across this junction. *Double stranded* instructs software to apply sequence searches to both strands (i.e., sequence and reverse complement of sequence).

| Nucleic Acid Topology | URI for Nucleic Acid Topology Term in SO |
|---|---|
| linear | http://identifiers.org/so/SO:0000987 |
| circular | http://identifiers.org/so/SO:0000988 |
| single-stranded | http://identifiers.org/so/SO:0000984 |
| double-stranded | http://identifiers.org/so/SO:0000985 |

Table 3: Sequence Ontology (SO) terms to encode DNA or RNA topology information in the type properties of a Component.

#### *The `role` property*

A `Component` MAY have any number of `role` properties, each of type `URI`, that MUST identify terms from ontologies that are consistent with the `type` property of the `Component`. For example, the `role` property of a DNA or RNA `Component` could contain URIs identifying terms from the Sequence Ontology (SO). As a best practice, a DNA or RNA `Component` SHOULD contain exactly one `URI` that refers to a term from the sequence feature branch of the SO. Similarly, the role properties of a protein and simple chemical `Component` SHOULD respectively contain `URI`s identifying terms from the `MolecularFunction` (`GO:0003674`) branch of the Gene Ontology (GO) and the **`role`** (`CHEBI:50906`) branch of the CHEBI ontology. Table 4 contains a partial list of possible ontology terms for the `role` properties and their `URI`s. These terms are organized by the type of `Component` to which they SHOULD apply (see Table 2). Any `Component` that can be well-described by one of the terms in Table 4 MUST use the `URI` for that term as a `role`.

These `URI`s might identify descriptive biological roles, such as "metabolic pathway" and "signaling cascade," but they can also identify identify "logical" roles, such as "inverter" or "AND gate", or other abstract roles for describing the function of design. Interpretation of the meaning of such roles currently depends on the software tools that read and write them.

| Component Role | URI for Ontology Term | Component Type |
|---|---|---|
| Promoter | `http://identifiers.org/so/SO:0000167` | DNA |
| RBS | `http://identifiers.org/so/SO:0000139` | DNA |
| CDS | `http://identifiers.org/so/SO:0000316` | DNA |
| Terminator | `http://identifiers.org/so/SO:0000141` | DNA |
| Gene | `http://identifiers.org/so/SO:0000704` | DNA |
| Operator | `http://identifiers.org/so/SO:0000057` | DNA |
| Engineered Gene | `http://identifiers.org/so/SO:0000280` | DNA |
| mRNA | `http://identifiers.org/so/SO:0000234` | RNA |
| Effector | `http://identifiers.org/chebi/CHEBI:35224` | Small Molecule |
| Transcription Factor | `http://identifiers.org/go/GO:0003700` | Protein |

Table 4: Partial list of ontology terms to specify the `role` property of a `Component`, organized by the type of `Component` to which they are intended to apply (see Table 2).

#### *The `hasSequence` property*

A `Component` MAY have any number of `hasSequence` properties, each of type `URI`, that MUST reference a `Sequence` object (see Section 6.3). These objects define the primary structure or structures of the `Component`.

If a `Feature` of a `Component` refers to a `Location`, and this `Location` refers to a `Sequence`, then the `Component` MUST also include a `hasSequence` property that refers to this `Sequence`.

Many `Component` objects will have exactly one `hasSequence` property that refers to a `Sequence` object. In this case, if its has a `type` from Table 2 and there is an `encoding` that is cross-listed with this term in Table 1, then the `Sequence` objects MUST have this encoding (e.g., a `Component` of `type` DNA must have a `Sequence` with an IUPAC DNA `encoding`). This `Sequence` is implicitly the entire sequence for this `Component` (In other words, it is equivalent to a `SequenceFeature` with an `EntireSequence` `Location` that refers to this `Sequence`).

#### *The `hasFeature` property*

A `Component` MAY have any number of `hasFeature` properties, each of type `URI` that MUST reference a `Feature` object (see Section 6.4.1). The set of relations between `Feature` and `Component` objects MUST be strictly acyclic.

Taking the `Component` class as analogous to a blueprint or specification sheet for a biological part or a system of interacting biological elements, the `Feature` class represents the specific occurrence of a part, subsystem, or other notable aspect within that design. This mechanism also allows a biological design to include multiple instances of a particular part (defined by reference to the same Component). For example, the Component of a polycistronic gene could contain two SubComponent objects that refer to the same Component of a CDS. As another example, consider the Component for a network of two-input repressor devices in which the particular repressors have not yet been chosen. This Component could contain multiple SubComponent objects that refer to the same Component of an abstract two-input repressor device.

The hasFeature properties of Component objects can be used to construct a hierarchy of SubComponent and Component objects. If a Component in such a hierarchy refers to a Location object, and there exists a Component object lower in the hierarchy that refers to a Location object that refers to the same Sequence with the same encoding, then the elements properties of these Sequence objects SHOULD be consistent with each other, such that well-defined mappings exist from the "lower level" elements to the "higher level" elements in accordance with their shared encoding properties. This mapping is also subject to any restrictions on the positions of the Feature objects in the hierarchy that are imposed by the SubComponent, SequenceFeature, or Constraint objects contained by the Component objects in the hierarchy.

For example, in a plasmid Component with a promoter SubComponent, the sequence at the promoter's Location within the plasmid should be the sequence for the promoter. More concretely, consider DNA Component that refers to a Sequence with an IUPAC DNA encoding and an elements String of "gattaca." In turn, this Component could contain a SubComponent that refers to a "lower level" Component that also refers to a Sequence with an IUPAC DNA encoding. Consequently, a consistent elements String of this "lower level" Sequence could be "gatta," or perhaps "tgta" if the SubComponent is positioned by a Location with an orientation of "reverse complement" (see Section 6.4.2).

##### *The hasConstraint property*

A Component MAY have any number of hasConstraint properties, each of type URI, that MUST reference a Constraint object (see Section 6.4.3). These objects describe, among other things, any restrictions on the relative, sequence-based positions and/or orientations of the Feature objects contained by the Component, as well as spatial relations such as containment and identity relations. For example, the Component of a gene might specify that the position of its promoter SubComponent precedes that of its CDS SubComponent. This is particularly useful when a Component lacks a Sequence and therefore cannot specify the precise, sequence-based positions of its SubComponent objects using Location objects.

##### *The hasInteraction property*

A Component MAY have any number of hasInteraction properties, each of type URI, that MUST reference an Interaction object (see Section 6.4.4).

The Interaction class provides an abstract, machine-readable representation of behavior within a Component (whereas a more detailed model of the system might not be suited to machine reasoning, depending on its implementation). Each Interaction contains Participation objects that indicate the roles of the Feature objects involved in the Interaction.

##### *The hasInterface property*

A Component MAY have zero or one hasInterface property of type URI that MUST reference an Interface object (see Section 6.4.5).

An Interface object indicates the inputs, outputs, and non-directional points of connection to a Component.

##### *The hasModel property*

A Component MAY have any number of hasModel properties, each of type URI, that MUST reference a Model object (see Section 6.8).

Model objects are placeholders that link Component objects to computational models of any format. A Component object can link to more than one Model since each might encode system behavior in a different way or at a different level of detail.

#### 6.4.1 Feature

The Feature class, as shown in Figure 9 is used to compose Component objects into a structural or functional hierarchy.

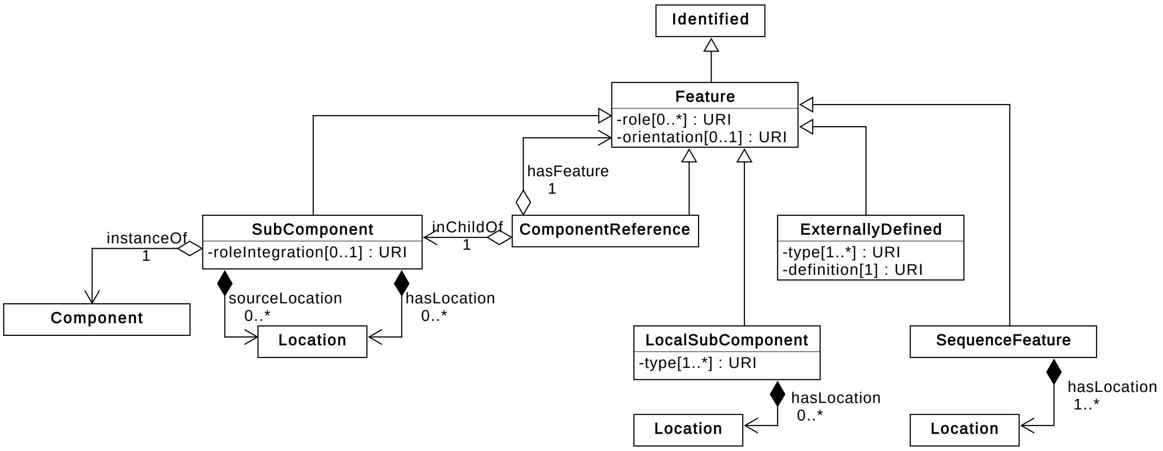

Figure 9: Diagram of the Feature class, its children, and associated properties.

##### *The* `role` *property*

Each Feature can have zero or more role property URIs describing the purpose or potential function of this Feature in the *context* of its parent Component. If the role for a SubComponent is left unspecified, then the role is determined by the role property of the Component that it is an instanceOf. If provided, these role property URIs MUST identify terms from appropriate ontologies. Roles are not restricted to describing biological function; they may annotate a Feature's function in any domain for which an ontology exists. A table of recommended ontology terms for role is given in Table 4.

It is RECOMMENDED that these role property URIs identify terms that are compatible with the type properties of the Feature's parent Component. For example, a role of a Feature which belongs to a Component of type DNA might refer to terms from the Sequence Ontology. Likewise, for any feature that is a SubComponent, the role SHOULD be compatible with the type of the Component that it links to through its instanceOf property.

##### *The* `orientation` *property*

The orientation property is OPTIONAL and has a data type of URI. This can be used to indicate how any associated double-stranded Feature is oriented on the elements of a Sequence from their parent Component. Table 5 provides a list of REQUIRED orientation URIs. If a Feature object has an orientation, then it MUST come from Table 5.

##### 6.4.1.1 SubComponent

The SubComponent class is a subclass of the Feature class that can be used to specify structural hierarchy. For example, the Component of a gene might contain four SubComponent objects: a promoter, RBS, CDS, and terminator, each linked to a Component that provides the complete definition. In turn, the Component of the promoter SubComponent might itself contain SubComponent objects defining various operator sites, etc.

| Orientation URI | Description |
|---|---|
| http://sbols.org/v2#inline | The region specified by this Feature or Location is on the elements of a Sequence. |
| http://sbols.org/v2#reverseComplement | The region specified by this Feature or Location is on the reverse-complement mapping of the elements of a Sequence. The exact nature of this mapping depends on the encoding of the Sequence. |

Table 5: REQUIRED URIs for the orientation property

##### *The roleIntegration property*

A roleIntegration specifies the relationship between a SubComponent instance's own set of role properties and the set of role properties on the included Component.

The roleIntegration property has a data type of URI. A SubComponent instance with zero role properties MAY OPTIONALLY specify a roleIntegration. A SubComponent instance with one or more role properties MUST specify a roleIntegration from Table 6. If zero SubComponent role properties are given and no SubComponent roleIntegration is given, then http://sbols.org/v2#mergeRoles is assumed. It is RECOMMENDED to specify SubComponent role values only if the result would differ from the role values belonging to this SubComponent's included Component.

| roleIntegration URI | Description |
|---|---|
| http://sbols.org/v2#overrideRoles | In the context of this SubComponent, ignore any role given for the included Component. Instead use only the set of zero or more role properties given for this SubComponent. |
| http://sbols.org/v2#mergeRoles | Use the union of the two sets: both the set of zero or more role properties given for this SubComponent as well as the set of zero or more role properties given for the included Component. |

Table 6: Each roleIntegration mode is associated with a rule governing how a SubComponent's role values are to be combined with the included Component's role values.

##### *The instanceOf property*

The instanceOf property is a REQUIRED URI that refers to the Component providing the definition for this SubComponent. Among other things, as described in the previous section, this Component effectively provides information about the type and role of the SubComponent.

The instanceOf property MUST NOT refer to the same Component as the one that contains the SubComponent. Furthermore, SubComponent objects MUST NOT form a cyclical chain of references via their instanceOf properties and the Component objects that contain them. For example, consider the SubComponent objects $A$ and $B$ and the Component objects $X$ and $Y$. The reference chain "$X$ has feature $A$, $A$ is an instance of $Y$, $Y$ has feature $B$, and $B$ is an instance of $X$" is cyclical.

##### *The hasLocation property*

A SubComponent MAY have any number of hasLocation properties, each of type URI, that MUST refer to Location objects that indicates the location of the Sequence from the instanceOf Component in a Sequence of the parent Component.

If any hasLocation is defined, then there MUST BE precisely one Sequence in the instanceOf Component, as otherwise this relationship is ill-defined.

If no hasLocation is defined, this indicates a part / sub-part relationship for which sequence details have not (yet) been determined or involving types for which sequence relationships are not relevant (e.g., inclusion of a reaction chain within a larger metabolic network).

Allowing multiple Location objects on a single SubComponent is intended to enable representation of discontinuous regions (for example, a coding sequence encoded across a set of exons with interspersed introns). As such, the Location objects of a single SubComponent MUST NOT specify overlapping regions, since it is not clear what this would mean. There is no such concern with different objects, however, which can freely overlap in Location (for example, specifying overlapping linkers for sequence assembly).

###### *The **sourceLocation** property*

The sourceLocation property allows for only a portion of a Component's Sequence to be included, rather than its entirety. For example, when composing parts with certain assembly methods, some bases on the boundary may be removed or replaced. Another example is describing a deletion or replacement of a portion of a sequence.

A SubComponent MAY have any number of sourceLocation properties, each of type URI, that MUST refer to Location objects that indicate which elements of the instanceOf Component's Sequence are used in defining the parent of the SubComponent.

If there are no sourceLocation properties, then the whole Sequence is assumed to be included.

##### *6.4.1.2 ComponentReference*

The ComponentReference class is a subclass of Feature that can be used to reference Features within SubComponents.

###### *The **inChildOf** property*

The inChildOf property is a REQUIRED URI that refers to a SubComponent. The inChildOf property MUST refer to a SubComponent pointed directly to by the parent of the ComponentReference. Specifically:

- If the parent of the ComponentReference is a Component, then inChildOf MUST be one of its SubComponents.
- If the parent of the ComponentReference is another ComponentReference, then inChildOf MUST be a SubComponent of the Component linked as instanceOf the parent's inChildOf SubComponent.

###### *The **hasFeature** property*

The hasFeature property is a REQUIRED URI that refers to a Feature.

This can be used to either link to the Feature being referenced or to chain hierarchically through additional layers of SubComponent.

- If the Feature is a ComponentReference, then that ComponentReference acts as a hierarchical link in a chain of references, and MUST be either a child of the ComponentReference linking to it via hasFeature or a child of the Component linked as instanceOf the ComponentReference's inChildOf SubComponent.
- Otherwise, if the hasFeature refers to any other type of Feature, that Feature MUST be a child of the Component linked as instanceOf the ComponentReference's inChildOf SubComponent.

For example, ComponentReference R1 looking into a SubComponent for a plasmid might link with hasFeature to its own child ComponentReference R2, which in turn looks within the Component defining the plasmid to the plasmid's CDS SubComponent, in turn using hasFeature to reference a SequenceFeature within the Component that defines that CDS.

##### 6.4.1.3 LocalSubComponent

The LocalSubComponent class is a subclass of Feature. This class serves as a way to create a placeholder in more complex Components, such as a variable to be filled in later or a composite that exists only within the context of the parent Component.

###### The hasLocation property

A LocalSubComponent MAY have any number of hasLocation properties, each of type URI, that MUST refer to Location objects. These follow the same restrictions as for the hasLocation of a SubComponent, notably that the Locations of hasLocation properties attached to the same LocalSubComponent MUST NOT overlap.

###### The type property

The type property is REQUIRED and contains one or more URIs. The type property is identical to its use in Component.

##### 6.4.1.4 ExternallyDefined

The ExternallyDefined class has been introduced so that external definitions in databases like ChEBI or UniProt can be referenced.

###### The type property

The type property is REQUIRED and contains one or more URIs. The type property is identical to its use in Component.

###### The definition property

The definition property is REQUIRED and is of type URI that links to a canonical definition external to SBOL. When possible, such definitions SHOULD use the recommended external resources in Section 7.6. For example, an ExternallyDefined simple chemical might link to ChEBI and a protein might link to UniProt.

##### 6.4.1.5 SequenceFeature

The SequenceFeature class describes one or more regions of interest on the Sequence objects referred to by its parent Component.

###### The hasLocation property

A SequenceFeature MAY have any number of hasLocation properties, each of type URI, that MUST refer to Location objects. These follow the same restrictions as for the hasLocation of a SubComponent, notably that the Locations of hasLocation properties attached to the same SequenceFeature MUST NOT overlap.

#### 6.4.2 Location

The Location class (as shown in Figure 10) is used to represent the location of Features within Sequences. This class is extended by the Range, Cut, and EntireSequence classes

###### The orientation property

The orientation property is OPTIONAL and has a data type of URI. All subclasses of Location share this property, which can be used to indicate how any associated double-stranded Feature is oriented on the elements of a Sequence from their parent Component. Table 5 provides a list of REQUIRED orientation URIs. If a Location object has an orientation, then it MUST come from Table 5.

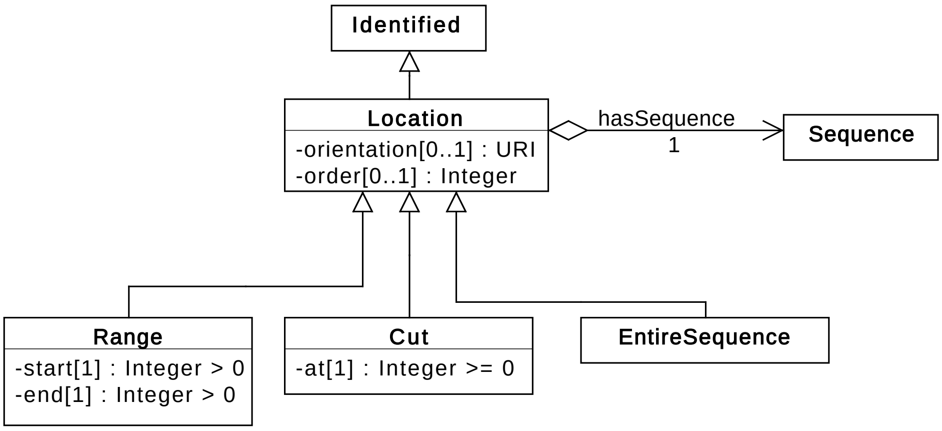

Figure 10: Diagram of the Location class and its associated properties.

##### *The order property*

The order property is OPTIONAL and has a data type of Integer. If there are multiple Location objects associated with a Feature, the order property is used to specify the order (in increasing value) in which the specified Locations are to be joined to form the sequence of the Feature. Note that order values MAY be non-sequential and non-positive, if desired.

##### *The hasSequence property*

The hasSequence property is REQUIRED and MUST contain the URI of a Sequence object. All subclasses of Location share this property, which indicates which Sequence object referenced by the containing Component is referenced by the Location.

#### 6.4.2.1 Range

A Range object specifies a region via discrete, inclusive start and end positions that correspond to indices for characters in the elements String of a Sequence.

Note that the index of the first location is 1, as is typical practice in biology, rather than 0, as is typical practice in computer science.

##### *The start property*

The start property specifies the inclusive starting position of the Range. This property is REQUIRED and MUST contain an Integer value greater than zero.

##### *The end property*

The end property specifies the inclusive ending position of the Range. This property is REQUIRED and MUST contain an Integer value greater than zero. In addition, this Integer value MUST be greater than or equal to that of the start property.

#### 6.4.2.2 Cut

The Cut class has been introduced to enable the specification of a region between two discrete positions. This specification is accomplished using the at property, which specifies a discrete position that corresponds to the index of a character in the elements String of a Sequence (except in the case when at is equal to zero—see below).

##### *The `at` property*

The `at` property is REQUIRED and MUST contain an `Integer` value greater than or equal to zero. The region specified by the `Cut` is between the position specified by this property and the position that immediately follows it. When the `at` property is equal to zero, the specified region is immediately before the first discrete position or character in the `elements` `String` of a `Sequence`.

##### 6.4.2.3 EntireSequence

The `EntireSequence` class does not have any additional properties. Use of this class indicates that the linked Sequence describes the entirety of the `Component` or `Feature` parent of this Location object.

#### 6.4.3 Constraint

The `Constraint` class can be used to assert restrictions on the relationships of pairs of `Feature` objects contained by the same parent `Component`. Uses of this class include expressing containment (e.g., a plasmid transformed into a chassis strain), identity mappings (e.g., replacing a placeholder value with a complete definition), and expressing relative, sequence-based positions (e.g., the ordering of features within a template). Each `Constraint` includes the `subject`, `object`, and `restriction` properties.

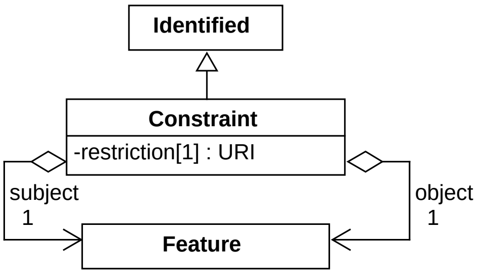

Figure 11: Diagram of the `Constraint` class and its associated properties.

##### *The `subject` property*

The `subject` property is REQUIRED and MUST contain a `URI` that refers to a `Feature` contained by the same parent `Component` that contains the `Constraint`.

##### *The `object` property*

The `object` property is REQUIRED and MUST contain a `URI` that refers to a `Feature` contained by the same parent `Component` that contains the `Constraint`. This `Feature` MUST NOT be the same `Feature` that the `Constraint` refers to via its `subject` property.

##### *The `restriction` property*

The `restriction` property is REQUIRED and has a data type of `URI`. This property MUST indicate the type of restriction on the locations, orientations, or identities of the `subject` and `object` `Feature` objects in relation to each other. The `URI` value of this property SHOULD come from the RECOMMENDED `URI`s in Table 7, Table 8, and Table 9.

| Restriction URI | Description |
|---|---|
| http://sbols.org/v3#verifyIdentical | The subject and object, after tracing through any layers of ComponentReference, MUST both refer to SubComponent objects with the same instanceOf value or both refer to ExternallyDefined objects with the same definition. *Example: a promoter included via two different subsystems must be the identical.* |
| http://sbols.org/v3#differentFrom | The subject and object, after tracing through any layers of ComponentReference, MUST NOT both refer to SubComponent objects with the same instanceOf value or both refer to ExternallyDefined objects with the same definition. *Example: two fluorescent reporters must be different.* |
| http://sbols.org/v3#replaces | In the context of the parent object of the Constraint, information about the subject should be used in place of all instances of the object. *Example: the J23101 promoter replaces a generic promoter.* |
| http://sbols.org/v3#sameOrientationAs | The subject and object Component objects MUST have the same orientation. *Example: a promoter has the same orientation as the coding sequence it controls.* |
| http://sbols.org/v2#oppositeOrientationAs | The subject and object Component objects MUST have opposite orientations. *Example: a promoter has the opposite orientation as an invertase-activated coding sequence it controls.* |

Table 7: RECOMMENDED URIs for expressing identity and orientation with the restriction property.

| Restriction URI | Description |
|---|---|
| http://sbols.org/v3#isDisjointFrom | The subject and object do not overlap in space. *Example: a plasmid is disjoint from a chromosome.* |
| http://sbols.org/v3#strictlyContains | The subject entirely contains the object: they do not share a boundary. *Example: a cell contains a plasmid* |
| http://sbols.org/v3#contains | The subject contains the object and they might or might not share a boundary (i.e., union of strictlyContains, equals, and covers. *Example: a cell contains a protein that may or may not bind to its membrane.* |
| http://sbols.org/v3#equals | The subject and object occupy the same location in space. *Example: a small molecule is distributed throughout an entire sample.* |
| http://sbols.org/v3#meets | The subject and object are connected at a shared boundary. *Example: two strains of adherent cells meet at their membranes.* |
| http://sbols.org/v3#covers | The subject contains the object but also shares a boundary. *Example: a cell covers its transmembrane proteins.* |
| http://sbols.org/v3#overlaps | The subject and object overlap in space, but portions of each are outside of the other. *Example: a transmembrane protein overlaps the cell membrane.* |

Table 8: RECOMMENDED URIs for expressing topological relations with the restriction property.

#### 6.4.4 Interaction

The Interaction class (as shown in Figure 12) provides more detailed description of how the Feature objects of a Component are intended to work together. For example, this class can be used to represent different forms of genetic regulation (e.g., transcriptional activation or repression), processes from the central dogma of biology (e.g. transcription and translation), and other basic molecular interactions (e.g., non-covalent binding or enzymatic phosphorylation). Each Interaction includes type properties that refer to descriptive ontology terms and hasParticipation properties that describe which Feature objects participate in which ways in the Interaction.

| Restriction URI | Description |
|---|---|
| http://sbols.org/v3#precedes | The start of the location for subject is less than the start of the location for object (i.e., union of strictlyPrecedes, meets, and overlaps). *Example: a promoter precedes a ribosome entry site, but the exact boundary between the two will be determined by sequence optimization and assembly planning.* |
| http://sbols.org/v3#strictlyPrecedes | The end of the location for subject is less than the start of the location for object. *Example: a promoter strictly precedes a terminator (with a CDS between them).* |
| http://sbols.org/v3#meets | The end of the location for subject is equal to the start of the location for object. Note: this is a stronger interpretation of meets from Table 8 in the context of a linear sequence. *Example: the 3' region adjacent to a blunt restriction site meets the 5' region adjacent to the site.* |
| http://sbols.org/v3#overlaps | The start of the location for subject is before the start of the location for object and the end of the location for subject is before the end of the location for object. Note: this is a stronger interpretation of overlaps from Table 8 in the context of a linear sequence. *Example: two adjacent oligos overlap in a Gibson assembly plan.* |
| http://sbols.org/v3#contains | The start of the location for subject is less than or equal to the start of the location for object and the end of the location for subject is greater than or equal to the end of the location for object (i.e., union of strictlyContains, equals, finishes, and starts). Note: this is a stronger interpretation of contains from Table 8 in the context of a linear sequence. *Example: a composite part contains a promoter.* |
| http://sbols.org/v3#strictlyContains | The start of the location for subject is before the start of the location for object and the end of the location for subject is after the end of the location for object. Note: this is a stronger interpretation of strictlyContains from Table 8 in the context of a linear sequence. *Example: an RNA transcript strictly contains an intron.* |
| http://sbols.org/v3#equals | The start and end of the location for subject are equal to the start and end of the location for object. Note: this is a stronger interpretation of equals from Table 8 in the context of a linear sequence. *Example: the transcribed region of a CDS part equals the entire part.* |
| http://sbols.org/v3#finishes | The start of the location for subject is after the start of the location for object and the end of the location for subject is equal to the end of the location for object. *Example: a terminator finishes an expression cassette.* |
| http://sbols.org/v3#starts | The start of the location for subject is equal to the start of the location for object and the end of the location for subject is before the end of the location for object. *Example: a promoter starts an expression cassette.* |

Table 9: RECOMMENDED URIs for expressing sequential relations with the restriction property.

##### *The type property*

An Interaction is REQUIRED to have one or more type properties, each of type URI, that describes the behavior represented by an Interaction.

Each type property MUST identify terms from appropriate ontologies. It is RECOMMENDED that exactly one URI specified by a type property refer to a term from the occurring entity branch of the Systems Biology Ontology (SBO). Table 10 provides a partial list of possible SBO terms for the type property and their corresponding URIs.

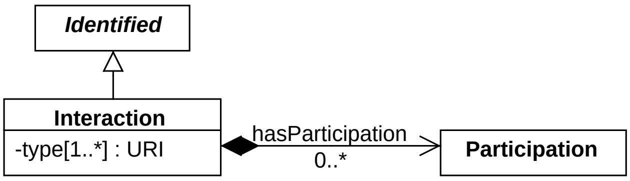

Figure 12: Diagram of the Interaction class and its associated properties.

| Interaction Type | URI for SBO Term |
|---|---|
| Inhibition | http://identifiers.org/biomodels.sbo/SBO:0000169 |
| Stimulation | http://identifiers.org/biomodels.sbo/SBO:0000170 |
| Biochemical Reaction | http://identifiers.org/biomodels.sbo/SBO:0000176 |
| Non-Covalent Binding | http://identifiers.org/biomodels.sbo/SBO:0000177 |
| Degradation | http://identifiers.org/biomodels.sbo/SBO:0000179 |
| Genetic Production | http://identifiers.org/biomodels.sbo/SBO:0000589 |
| Control | http://identifiers.org/biomodels.sbo/SBO:0000168 |

Table 10: Partial list of SBO terms to specify the type property of an Interaction.

If an Interaction is well described by one of the terms from Table 10, then a type property MUST refer to the URI that identifies this term. Lastly, if there are multiple type properties for an Interaction, then they MUST identify non-conflicting terms. For example, the SBO terms "stimulation" and "inhibition" would conflict.

##### *The **hasParticipation** property*

An Interaction MAY have any number of hasParticipation properties, each of type URI, that MUST reference a Participation object, each of which identifies the role that its referenced Feature plays in the Interaction.

Even though an Interaction generally contains at least one Participation, the case of zero Participation objects is allowed because it is plausible that a designer might want to specify that an Interaction will exist, even if its participants have not yet been determined.

#### 6.4.4.1 Participation

Each Participation (see Figure 13) represents how a particular Feature behaves in its parent Interaction.

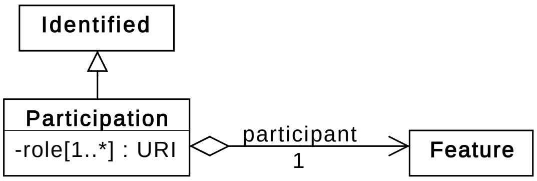

Figure 13: Diagram of the Participation class and its associated properties.

##### *The `role` property*

A `Participation` is REQUIRED to have one or more `role` properties, each of type `URI`, that describes the behavior of a `Participation` (and by extension its referenced `Feature`) in the context of its parent `Interaction`.

Each `role` property MUST identify terms from appropriate ontologies. It is RECOMMENDED that exactly one `URI` specified by a `role` property refer to a term from the participant role branch of the SBO. Table 11 provides a partial list of possible SBO terms for the `role` properties and their corresponding `URI`s.

| Participation Role | URI for SBO Term | Interaction Types |
|---|---|---|
| Inhibitor | http://identifiers.org/biomodels.sbo/SBO:0000020 | Inhibition |
| Inhibited | http://identifiers.org/biomodels.sbo/SBO:0000642 | Inhibition |
| Stimulator | http://identifiers.org/biomodels.sbo/SBO:0000459 | Stimulation |
| Stimulated | http://identifiers.org/biomodels.sbo/SBO:0000643 | Stimulation |
| Reactant | http://identifiers.org/biomodels.sbo/SBO:0000010 | Non-Covalent Binding, Degradation Biochemical Reaction |
| Product | http://identifiers.org/biomodels.sbo/SBO:0000011 | Non-Covalent Binding, Genetic Production, Biochemical Reaction |
| Promoter | http://identifiers.org/biomodels.sbo/SBO:0000598 | Inhibition, Stimulation, Genetic Production |
| Modifier | http://identifiers.org/biomodels.sbo/SBO:0000019 | Biochemical Reaction, Control |
| Modified | http://identifiers.org/biomodels.sbo/SBO:0000644 | Biochemical Reaction, Control |
| Template | http://identifiers.org/biomodels.sbo/SBO:0000645 | Genetic Production |

Table 11: Partial list of SBO terms to specify the `role` properties of a `Participation`.

If a `Participation` is well described by one of the terms from Table 11, then a `role` property MUST refer to the `URI` that identifies this term. Also, if a `Participation` belongs to an `Interaction` that has a type listed in Table 10, then the `Participation` SHOULD have a role that is cross-listed with this type in Table 11. Lastly, if there are multiple `role` properties for a `Participation`, then they MUST identify non-conflicting terms. For example, the SBO terms “stimulator” and “inhibitor” would conflict.

##### *The `participant` property*

The `participant` property MUST specify precisely one `Feature` object that plays the designated role in its parent `Interaction` object.

### 6.4.5 Interface

The `Interface` class (shown in Figure 14) is a way of explicitly specifying the interface of a `Component`.

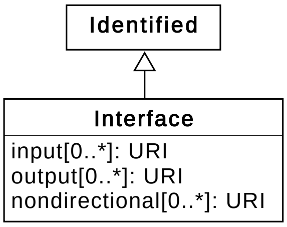

Figure 14: Diagram of the `Interface` class and its associated properties.

##### *The input property*

An Interface MAY have any number of input properties, each of type URI, that MUST reference a Feature object in the same Component.

##### *The output property*

An Interface MAY have any number of output properties, each of type URI, that MUST reference a Feature object in the same Component.

##### *The nondirectional property*

An Interface MAY have any number of nondirectional properties, each of type URI, that MUST reference a Feature object in the same Component. Note that nondirectional can imply both bidirectional as well as situations where there are no flows (for instance – a physical interface).

### 6.5 CombinatorialDerivation

The purpose of the CombinatorialDerivation class is to specify combinatorial biological designs without having to specify every possible design variant. For example, a CombinatorialDerivation can be used to specify a library of reporter gene variants that include different promoters and RBSs without having to specify a Component for every possible combination of promoter, RBS, and CDS in the library. Component objects that realize a CombinatorialDerivation can be derived in accordance with the class properties template, hasVariableComponent, and strategy (see Figure 15).

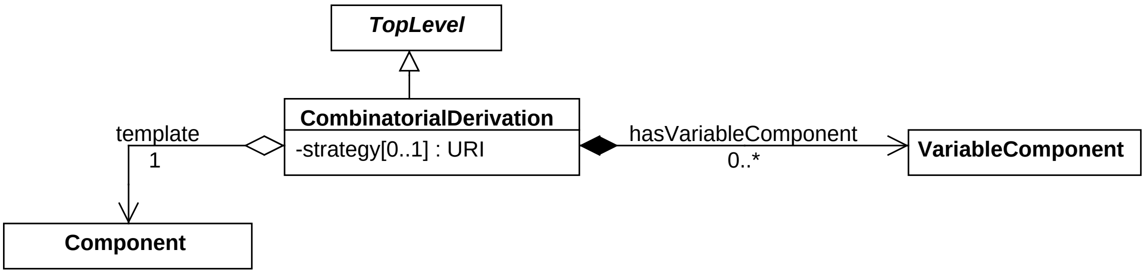

Figure 15: Diagram of the CombinatorialDerivation class and its associated properties.

##### *The template property*

The template property is REQUIRED and MUST contain a URI that refers to a Component. This Component is expected to serve as a template for the derivation of new Component objects. Consequently, its hasFeature properties SHOULD contain one or more SubComponent objects that describe its substructure (referred to hereafter as template SubComponent objects), and its hasConstraint property MAY also contain one or more Constraint objects that constrain this substructure.

When a Component is derived in accordance with a CombinatorialDerivation, the prov:wasDerivedFrom property of the derived Component SHOULD refer to the CombinatorialDerivation. When multiple Component objects are derived in accordance with the same CombinatorialDerivation, they MAY be referred to by the member property of a Collection, in which case the prov:wasDerivedFrom property of the Collection SHOULD also refer to this CombinatorialDerivation.

If the type property of the template Component contains one or more URIs, then the type property of any derived Component SHOULD also contain those URIs. The same holds true for the role properties of these Component objects.

***The hasVariableComponent property***

Each VariableComponent child of a CombinatorialDerivation defines the set of possible values for one of the variables in the template. A CombinatorialDerivation object can have zero or more hasVariableComponent properties, each of type URI, specifying a VariableComponent. The set of hasVariableComponent properties MUST NOT contain two or more VariableComponent objects that refer to the same template SubComponent via their variable properties (i.e., do not define the same variable twice).

The variable properties of VariableComponent objects control which SubComponent objects in the template are modified in a derived Component. If no variable property of one of these VariableComponent objects refers to a template SubComponent, then it is not a variable and the derived object SHOULD have a SubComponent with identical instanceOf and sourceLocation properties and a prov:wasDerivedFrom property that refers to the template SubComponent.

If a SubComponent in the template is referred to by some variable in a VariableComponent, then it is a variable and it SHOULD be replaced in the derived Component by a number of SubComponent objects constrained by the number specified by the cardinality property of the VariableComponent (see Table 13). Each instanceOf property of such a SubComponent object in the derived Component MUST refer to a Component object specified by a variant, contained within a variantCollection, or derived from a variantDerivation of the VariableComponent.

Finally, all derived SubComponent objects MUST follow the restriction properties of any Constraint objects that refer to their corresponding template SubComponent, and SHOULD have values of role that contain the same values as the role in the template SubComponent.

***The strategy property***

The strategy property is OPTIONAL and has a data type of URI. Table 12 provides a list of REQUIRED strategy URIs. If the strategy property is not empty, then it MUST contain a URI from Table 12. This property recommends how many Component objects SHOULD be derived from the template Component.

| Strategy URI | Description |
| --- | --- |
| http://sbols.org/v2#enumerate | Derivation SHOULD produce all possible Component objects specified by the CombinatorialDerivation. |
| http://sbols.org/v2#sample | Derivation SHOULD produce a subset of possible Component objects specified by CombinatorialDerivation. The manner in which this subset is chosen is left unspecified. |

Table 12: REQUIRED URIs for the strategy property.

#### 6.5.1 VariableComponent

As described above, the VariableComponent class specifies a variable and set of values that will replace one of the SubComponent objects in the template of a CombinatorialDerivation. The variable is specified by the variable property, and the set of values is defined by the union of Component objects referred to by the variant, variantCollection, and variantDerivation properties.

Note that this union is intended to be a set and not a multi-set. For example, if the variant property contains a Component $A$ and the variantCollection property has a Collection containing both Component $A$ and Component $B$, then $A$ SHOULD NOT be selected twice during enumeration, and it SHOULD NOT be selected twice as much as $B$ during sampling.

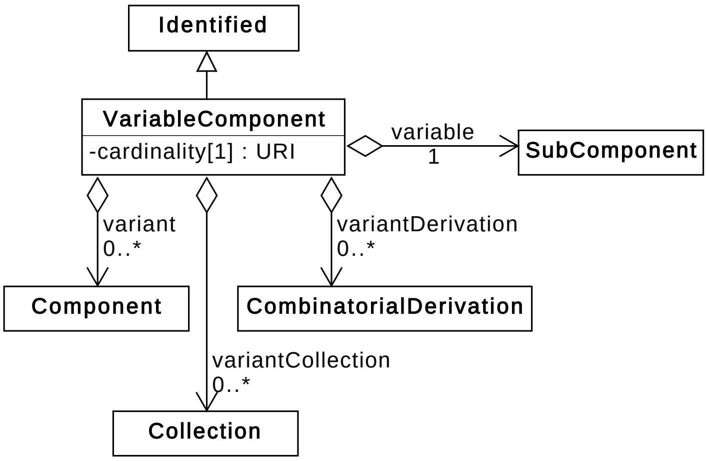

Figure 16: Diagram of the VariableComponent class and its associated properties.

#### The variable property

The variable property is REQUIRED and MUST contain a URI that refers to a template SubComponent in the template Component referred to by this VariableComponent's parent CombinatorialDerivation

#### The variant property

A VariableComponent object can have zero or more variant properties, each of type URI, specifying a Component object. This property specifies individual Component objects to serve as options when deriving a new SubComponent for the variable SubComponent from the template.

#### The variantCollection property

A VariableComponent object can have zero or more variantCollection properties, each of type URI, specifying a Collection object. Such a Collection MUST NOT contain any objects besides Component objects or Collection objects that themselves contain only Component or Collection objects. This property enables the specification of existing groups of Component objects to serve as options.

#### The variantDerivation property

A VariableComponent object can have zero or more variantDerivation properties, each of type URI, specifying a CombinatorialDerivation object. This property enables the specification of Component objects derived in accordance with another CombinatorialDerivation to serve as options when deriving a new SubComponent for the variable SubComponent from the template. The variantDerivation properties of a VariableComponent MUST NOT refer to the CombinatorialDerivation that contains this VariableComponent. Furthermore, such VariableComponent objects MUST NOT form a cyclical chain of references via their variantDerivation properties and the CombinatorialDerivation objects that contain them.

#### The cardinality property

The cardinality property is REQUIRED and has type of URI. This property specifies how many SubComponent objects SHOULD be derived from the template SubComponent during the derivation of a new Component. The URI value of this property MUST come from the URIs provided in Table 13.

| Cardinality URI | Description |
|---|---|
| http://sbols.org/v2#zeroOrOne | No more than one SubComponent in the derived Component SHOULD have a prov:wasDerivedFrom property that refers to the template SubComponent. |
| http://sbols.org/v2#one | Exactly one SubComponent in the derived Component SHOULD have a prov:wasDerivedFrom property that refers to the template SubComponent. |
| http://sbols.org/v2#zeroOrMore | Any number of SubComponent objects in the derived Component MAY have prov:wasDerivedFrom properties that refer to the template SubComponent. |
| http://sbols.org/v2#oneOrMore | At least one SubComponent in the derived Component SHOULD have a prov:wasDerivedFrom property that refers to the template SubComponent. |

Table 13: REQUIRED URIs for the cardinality property.

### 6.6 Implementation

An Implementation represents a realized instance of a Component, such a sample of DNA resulting from fabricating a genetic design or an aliquot of a specified reagent. Importantly, an Implementation can be associated with a laboratory sample that was already built, or that is planned to be built in the future. An Implementation can also represent virtual and simulated instances. An Implementation may be linked back to its original design using the prov:wasDerivedFrom property inherited from the Identified superclass. An Implementation may also link to a Component that specifies its realized structure and/or function.

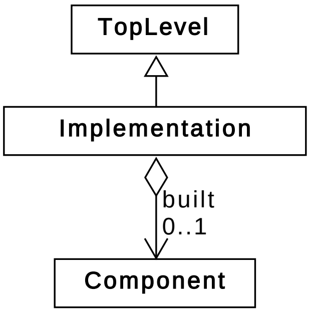

Figure 17: Diagram of the Implementation class and its associated properties.

##### *The built property*

The built property is OPTIONAL and MAY contain a URI that MUST refer to a Component. This Component is intended to describe the actual physical structure and/or functional behavior of the Implementation. When the built property refers to a Component that is also linked to the Implementation via PROV-O properties such as prov:wasDerivedFrom (see Section A.1), it can be inferred that the actual structure and/or function of the Implementation matches its original design. When the built property refers to a different Component, it can be inferred that the Implementation has deviated from the original design. For example, the latter could be used to document when the DNA sequencing results for an assembled construct do not match the original target sequence.

### 6.7 ExperimentalData

The purpose of the ExperimentalData class is to aggregate links to experimental data files. An ExperimentalData is typically associated with a single sample, lab instrument, or experimental condition and can be used to describe the output of the test phase of a design-build-test-learn workflow. For an example of the latter, see Figure 28.

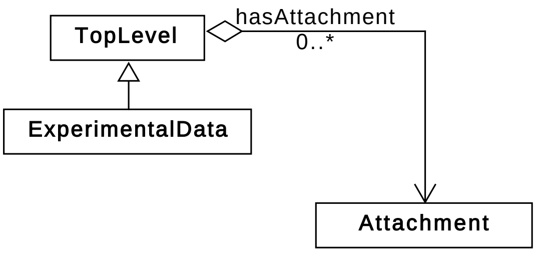

Figure 18: Diagram of the `ExperimentalData` class and its associated properties.

As shown in Figure 18, the `ExperimentalData` class aggregates links to experimental data files using the OPTIONAL `hasAttachment` property that it inherits from the `TopLevel` class.

### 6.8 Model

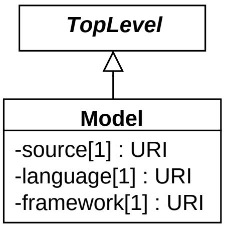

Figure 19: Diagram of the `Model` class and its associated properties.

The purpose of the `Model` class is to serve as a placeholder for an external computational model and provide additional meta-data to enable better reasoning about the contents of this model. In this way, there is minimal duplication of standardization efforts and users of SBOL can elaborate descriptions of `Component` function in the language of their choice.

The meta-data provided by the `Model` class include the following properties: the `source` or location of the actual content of the model, the `language` in which the model is implemented, and the model's `framework`.

##### *The `source` property*

The `source` property is REQUIRED and MUST contain a `URI` reference to the source file for a model.

##### *The `language` property*

The `language` property is REQUIRED and MUST contain a `URI` that specifies the language in which the model is implemented. It is RECOMMENDED that this `URI` refer to a term from the EMBRACE Data and Methods (EDAM) ontology. Table 14 provides a list of a few suggested languages from this ontology and their `URI`s. If the `language` property of a `Model` is well-described by one these terms, then it MUST contain the `URI` for this term as its value.

##### *The `framework` property*

The `framework` property is REQUIRED and MUST contain a `URI` that specifies the framework in which the model is implemented. It is RECOMMENDED this `URI` refer to a term from the modeling framework branch of the SBO

| Model Language | URI for EDAM Term |
|---|---|
| SBML | http://identifiers.org/edam/format_2585 |
| CellML | http://identifiers.org/edam/format_3240 |
| BioPAX | http://identifiers.org/edam/format_3156 |

Table 14: Terms from the EDAM ontology to specify the language property of a Model.

when possible. A few suggested modeling frameworks and their corresponding URIs are shown in Table 15. If the framework property of a Model is well-described by one these terms, then it MUST contain the URI for this term as its value.

| Framework | URI for SBO Term |
|---|---|
| Continuous | http://identifiers.org/biomodels.sbo/SBO:0000062 |
| Discrete | http://identifiers.org/biomodels.sbo/SBO:0000063 |

Table 15: SBO terms to specify the framework property of a Model.

### 6.9 Collection

The Collection class is a class that groups together a set of TopLevel objects that have something in common. Some examples of Collection objects:

- Results of a query to find all Component objects in a repository that function as promoters.
- A set of Component objects representing a library of genetic logic gates.
- A "parts list" for Component with a complex design, containing both that component and all of the Component, Sequence, and Model objects used to provide its full specification.

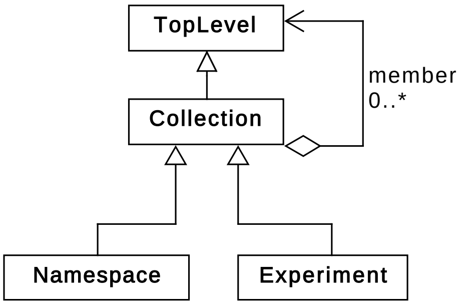

Figure 20: Diagram of the Collection class and its associated properties.

##### *The member property*

A Collection object can have zero or more member properties, each of type URI specifying a TopLevel object.

#### 6.9.1 Namespace

The Namespace class is a subclass of Collection and is used to define member entities that share the same URI prefix. Namely, all linked objects MUST have a URI prefix matching the URI of the Namespace object.

#### 6.9.2 Experiment

The purpose of the Experiment class is to aggregate ExperimentalData objects for subsequent analysis, usually in accordance with an experimental design. Namely, the member properties of an Experiment MUST refer to ExperimentalData objects.

### 6.10 Attachment

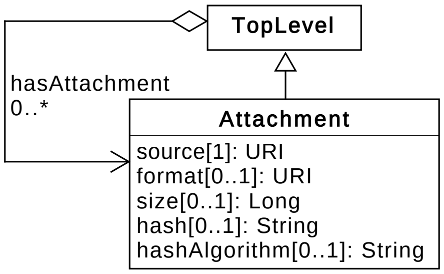

Figure 21: Diagram of the Attachment class and its associated properties.

The purpose of the Attachment class is to serve as a general container for data files, especially experimental data files. It provides a means for linking files and metadata to SBOL designs.

The meta-data provided by the Attachment class include the following properties: the source or location of the actual file of the attachment, the format of the file, the size of the file, and the hash for the file.

##### *The `source` property*

The source property is REQUIRED and MUST contain a URI reference to the source file.

##### *The `format` property*

The format property is OPTIONAL and MAY contain a URI that specifies the format of the attached file. It is RECOMMENDED that this URI refer to a term from the EMBRACE Data and Methods (EDAM) ontology.

##### *The `size` property*

The size property is OPTIONAL and MAY contain a long indicating the file size in bytes.

##### *The `hash` property*

The hash property is OPTIONAL and MAY contain a hash value for the file contents represented as a hexadecimal digest.

##### *The `hashAlgorithm` property*

The hashAlgorithm property is OPTIONAL and MAY contain the name of the hash algorithm used to generate the value of the hash property. The value of this property SHOULD be a hash name string from the IANA Named Information Hash Algorithm Registry, of which sha3-256 is currently RECOMMENDED. If the hash property is set, then hashAlgorithm MUST be set as well.

### 6.11 Annotation and Extension of SBOL

SBOL intentionally does not attempt to describe how all types of biological design data should be captured, since many of these data types (e.g., biological context and design performance metrics) are already covered by other standards, or lack a clear consensus on their proper representation, or are outside of the scope of SBOL.

SBOL is built upon the Resource Description Framework (RDF), and therefore can be used in conjunction with complementary standards as described in Section A. For example, use of the PROV-O ontology is recommended to capture provenance (see Section A.1). Additionally, user-defined RDF can be used in conjunction with SBOL objects to capture custom application-specific information that does not yet have a standardized representation. This annotation and extension mechanism is designed to enable new types of data to be easily incorporated into the SBOL standard once there is community consensus on their proper representation.

Several methods are supported for connecting the SBOL data model with other types of application-specific data:

- Custom data can be added to an SBOL object by annotating that object with non-conflicting properties. These properties could contain literal data types such as Strings or URIs that require a resolution mechanism to obtain external data. An example is annotating a Component with a property that contains a String description and URI for the parts registry from which its source data was originally imported.
- Custom data in the form of independent objects can participate in the SBOL data model if they are assigned one of the SBOL types Identified or TopLevel. An example is an RDF object that is annotated such that it represents a data sheet that describes the performance of a Component in a particular context.
- Finally, just as custom objects can be embedded in an SBOL document, external documents can embed or refer to SBOL objects. Support for this last case is not explicitly provided in this specification. Rather, this case depends on the external non-SBOL system managing its relationship to SBOL and data serialized in RDF, and is included here for completeness.

Each Identified object MAY be annotated with application-specific properties, which MUST be labelled using RDF predicates outside of the SBOL namespace. Additionally, application-specific types may be used in conjunction with the SBOL data model. These application-specific types MUST have two rdf:type properties: one type outside of the SBOL namespace AND an additional SBOL type of either:

- TopLevel, if the object is to be considered an SBOL top level (i.e., not owned by another object)
- Identified, if the object is not to be considered an SBOL top level (i.e., is owned by another object)

As with SBOL Identified objects, custom Identified objects (and thus also custom TopLevel objects) MAY also include the properties displayId, name, description, etc.

## 7 Recommended Best Practices

### 7.1 SBOL Versions

To differentiate between major versions of SBOL, different namespaces are used. For example, SBOL3 has the namespace `http://sbols.org/v3#`, while SBOL2 has the namespace `http://sbols.org/v2#`. These different versions of SBOL SHOULD NOT be semantically mixed. For example, an SBOL 3.x `SubComponent` SHOULD NOT refer to an SBOL 2.x `ComponentInstance`, and, likewise, an SBOL 2.x `ComponentInstance` SHOULD NOT refer to an SBOL 1.x `DnaComponent`.

### 7.2 Compliant SBOL Objects

Maintaining unique URIs for all SBOL objects can be challenging. To reduce this burden, users of SBOL 3.x are encouraged to follow a few simple rules when constructing the URIs and related properties for SBOL objects. When these rules are followed in constructing an SBOL object, we say that this object is *compliant*. These rules are as follows:

Compliant URIs for `TopLevel` objects MUST conform to the following pattern:

⟨namespace⟩/⟨collection_structure⟩/⟨displayId⟩

The ⟨namespace⟩ token MAY further decompose into ⟨domain⟩/⟨root⟩ tokens. The ⟨root⟩ and ⟨collection_structure⟩ tokens may optionally be omitted; alternatively, they may consist of an arbitrary number of delimiter-separated layers. Note that this pattern means that SBOL-compliant `URI`s can be automatically decomposed with the aid of a `Namespace`. SBOL-compliant objects can be easily remapped into new namespaces by changing only the ⟨namespace⟩.

Consider, for example, the SBOL-compliant `URI`:

"https://synbiohub.org/igem/2017_distribution/promoters/constitutive/BBa_J23101"

in `Namespace` "https://synbiohub.org/igem/2017_distribution". This `URI` can be decomposed as follows:

| | |
|---|---|
| namespace: | "https://synbiohub.org/igem/2017_distribution" |
| domain: | "https://synbiohub.org" |
| root: | "igem/2017_distribution" |
| collection: | "promoters/constitutive" |
| displayId: | "BBa_J23101" |

SBOL-compliant URIs also facilitate auto-construction of child objects with unique `URI`s. Child objects of `TopLevel` objects with compliant `URI`s MUST conform to the following pattern:
"⟨parent_uri⟩/⟨child_type⟩⟨child_type_counter⟩" where the ⟨parent_uri⟩ refers to the URI of the parent object, the ⟨child_type⟩ refers to the SBOL class of the child object, and ⟨child_type_counter⟩ is a unique index for the child object. The ⟨child_type_counter⟩ of a new object SHOULD be calculated at time of object creation as 1 + the maximum ⟨child_type_counter⟩ for each ⟨child_type⟩ object in the parent (e.g., "⟨parent_uri⟩/SequenceAnnotation37"). Note that numbering is independent for each type, so a `Component` can have children "SubComponent37" and "Constraint37".

All examples in this specification use compliant `URI`s.

### 7.3 Versioning SBOL Objects

SBOL 3.x does not specify an explicit versioning scheme. Rather it is left for experimentation across different tools. This allows version information to be included in the root (e.g., GitHub style: "igem/HEAD/"), collection structure (e.g., "promoters/constitutive/2/"), in tool-specific conventions on `displayId` (e.g., "BBa_J23101_v2") or in information outside of the `URI` (e.g., by attaching `prov:wasRevisionOf` properties).

### 7.4 Annotations: Embedded Objects vs. External References

When annotating an SBOL document with additional information, there are two general methods that can be used:

- Embed the information in the SBOL document using properties outside of the SBOL namespace.
- Store the information separately and annotate the SBOL document with `URI`s that point to it.

In theory, either method can be used in any case. (Note that a third case not discussed here is to annotate external objects with links to SBOL documents, rather than annotating SBOL documents with links to external objects.)

In practice, embedding large amounts of non-SBOL data into SBOL documents is likely to cause problems for people and software tools trying to manage and exchange such documents. Therefore, it is RECOMMENDED that small amounts of information (e.g., design notes or preferred graphical layout) be embedded in the SBOL model, while large amounts of information (e.g., the contents of the scientific publication from which a model was derived or flow cytometry data that characterizes performance) be linked with URIs pointing to external resources. The boundary between "small" and "large" is left deliberately vague, recognizing that it will likely depend on the particulars of a given SBOL application.

### 7.5 Completeness and Validation

RDF documents containing serialized SBOL objects might or might not be entirely self-contained. A SBOL document is self-contained or "complete" if every SBOL object referred to in the document is contained in the document. It is RECOMMENDED that serializations be complete whenever practical. In order words, when serializing an SBOL object, serialize all of the other objects that it points to, then serialize all of the other objects that these objects point to, etc., until the document is complete.

It is important to note that there is no guarantee that an RDF document contains valid SBOL. When SBOL objects are read from an RDF document, the program doing so SHOULD verify that all of the property values encoded therein have the correct data type (e.g., that the object pointed to by the `Sequence` property of a `Component` is really a `Sequence`). For complete files, this validation can be carried out entirely locally. For files that are not complete, an implementation either needs to have a means of validating those external references (e.g., by retrieving them from a repository), or it needs to mark them as unverified and not depend on their correctness.

### 7.6 Recommended Ontologies for External Terms

External ontologies and controlled vocabularies are an integral part of SBOL. SBOL uses `URI`s to access existing biological information through these resources. New SBOL-specific terms are defined only when necessary. For example, `Component` `type`s, such as DNA or protein, are described using Systems Biology Ontology (SBO) terms. Similarly, the `role`s of a DNA or RNA `Component` are described via Sequence Ontology (SO) terms. Although RECOMMENDED ontologies have been indicated in relevant sections where possible, other resources providing similar terms can also be used. A summary of these external sources can be found in Table 16.

The URIs for ontological terms SHOULD come from identifiers.org. However, it is acceptable to use terms from purl.org as an alternative, for example when RDF tooling requires URIs to be represented as compliant QNames. SBOL software may convert between these forms as required.

| SBOL Entity | Property | Preferred External Resource | More Information |
|---|---|---|---|
| **Component** | type | SBO (physical entity branch) | http://www.ebi.ac.uk/sbo/main/ |
| | type | SO (nucleic acid topology) | http://www.sequenceontology.org |
| | role | SO (*DNA* or *RNA*) | http://www.sequenceontology.org |
| | role | CHEBI (*small molecule*) | https://www.ebi.ac.uk/chebi/ |
| | role | UniProt (*protein*) | https://www.uniprot.org/ |
| | role | NCIT (*samples*) | https://ncithesaurus.nci.nih.gov/ |
| **Interaction** | type | SBO (occurring entity branch) | http://www.ebi.ac.uk/sbo/main/ |
| **Participation** | role | SBO (participant roles branch) | http://www.ebi.ac.uk/sbo/main/ |
| **Model** | language | EDAM | http://bioportal.bioontology.org/ontologies/EDAM |
| | framework | SBO (modeling framework branch) | http://www.ebi.ac.uk/sbo/main/ |
| **om:Measure** | type | SBO (systems description parameters) | http://www.ebi.ac.uk/sbo/main/ |

Table 16: Preferred external resources from which to draw values for various SBOL properties.

### 7.7 Annotating Entities with Date & Time

Entities in an SBOL document can be annotated with creation and modification dates. It is RECOMMENDED that predicates, or properties, from DCMI Metadata Terms SHOULD be used to include date and time information. The `created` and `modified` terms SHOULD respectively be used to annotate SBOL entities with creation and modification dates. Date and time values SHOULD be expressed using the XML Schema `DateTime` datatype (Biron et al., 2004). For example, "`2016-03-16T20:12:00Z`" specifies that the day is 16 March 2016 and the time is 20:12pm in UTC (Coordinated Universal Time).

### 7.8 Annotating Entities with Authorship information

Authorship information should ideally be added to `TopLevel` entities where possible. It is RECOMMENDED that the `creator` DCMI Metadata term SHOULD be used to annotate SBOL entities with authorship information using free text. This property can be repeated for each author.

### 7.9 Host Context / Ontologies for Experiments

#### *7.9.1 Mixtures via Components*

Any `Component` can be interpreted as specifying a mixture of the material entity (SBO:0000240) `Feature`s that it includes. The amount of each such instance included in the mixture SHOULD be specified by attaching a `om:Measure` with a `type` set to the appropriate SBO term. The SBO terms that are RECOMMENDED as appropriate are members of the Systems Description Parameter (SBO:0000545) branch of SBO. Examples include:

- SBO:0000540: fraction of an entity pool (e.g., 1/3 CHO cells, 2/3 HEK cells)
- SBO:0000472: molar concentration of an entity (e.g., 1 mM Arabinose)
- SBO:0000361: amount of an entity pool (e.g., 200 uL M9 media)

Mixtures MAY be defined recursively, as mixtures of mixtures of mixtures, etc.

#### *7.9.2 Media, Inducers, and Other Reagents*

Each reagent, whether "atomic" (e.g., rainbow bead control) or mixture (e.g., M9 media), SHOULD be represented as a `Component`.

The roles of reagents may vary in context: for example, Arabinose may serve as an inducer or as a media carbon source. As such, role SHOULD be indicated by an NCI Thesaurus (NCIT) term in a `role` property of the `SubComponent`. Examples include:

- NCIT:C64356: Positive Control
- NCIT:C48694: Cell
- NCIT:C85504: Media
- NCIT:C14419: Strain
- NCIT:C120268: Inducer

For more information on representing cells, strains, plasmids, and genomes, see Section 7.10.1

#### 7.9.3 Samples

A complete specification of a sample SHOULD be a `Component` that includes at least:

- A `SubComponent` instantiating each strain in the sample
- A `SubComponent` for the media or buffer
- A `SubComponent` for each additional reagent added to the media (e.g., inducers, antibiotics)
- `om:Measure`s on each of these specifying the amount in the sample
- `om:Measure`s on the `Component` for each environmental parameter (e.g., temperature, pH, culturing time)

#### 7.9.4 Other Experimental Parameters

In order to deal with parameters associated with the context in general but not specific instances, e.g., temperature, pH, total sample volume, the `hasMeasure` property of `Identified` can be used. The `hasMeasure` of a `Component` provides context-free information (e.g., the pH of M9 media, the GC-content of a GFP coding sequence), while the `hasMeasure` of a material entity (SBO:0000240) `Feature` provides a measurement in context (e.g., the dosage of Arabinose in a sample).

Values of these parameters SHOULD be specified by attaching a `om:Measure` with a `type` set to the appropriate SBO term. The SBO terms that are RECOMMENDED as appropriate are members of the Systems Description Parameter (SBO:0000545) branch of SBO. Examples include:

- SBO:0000147: thermodynamic temperature (e.g., culturing at 27 C)
- SBO:0000332: half-life of an exponential decay (e.g., decay rate of a gRNA)
- SBO:0000304: pH (e.g., pH of M9 media)

### 7.10 Multicellular System Designs

SBOL has been used extensively to represent designs in homogeneous systems, where the same design is implemented in every cell. However, in recent years there has been increasing interest in multicellular systems, where biological designs are split across multiple cells to optimize the system behavior and function. Therefore, there is a need to define a set of best practices so that multicellular systems can be captured using SBOL in a standard way.

#### 7.10.1 Representing Cell Types

To represent multicellular systems using SBOL, it is first necessary to represent cells. When doing so, it is important to be able to capture the following information: (i) taxonomy of the strain used, (ii) interactions occurring within cells of this type, and (iii) components inside the type of cell (e.g. genomes, plasmids). The approach RECOMMENDED in this section is capable of capturing this information, as shown in the example in Figure 22. It uses a `Component` to represent a system that contains cells of the given type. The cells themselves are represented by a `SubComponent` inside the `Component`, which is an `instanceOf` a `Component` capturing information about the species and strain of the cell in the design. This `Component` has a `type` of "cell" from the Gene Ontology (GO:0005623), and a `role` of "physical compartment" (SBO:0000290). Taxonomic information is captured by annotating the class instance with a URI for an entry in the NCBI Taxonomy Database.

As usual, other entities besides the cell that are relevant to the design are also captured as `Feature`s. When these are contained within the cell, they are captured using a `Constraint` with restriction `contains` with the cell as `subject` and contained object as `object`. Interactions which occur in this system are captured using the `Interaction` and `Participation` classes. Interactions which occur within the cell are specified by `Interaction` classes which contain the `SubComponent` instance representing the cell as a `participant` with a `role` of "physical compartment" (SBO:0000290).

#### 7.10.2 Multiple Cell Types in a Single Design

The same approach can be extended to represent systems with multiple types of cells. The multicellular system can be represented as a `Component` that includes each strain of cell as a `SubComponent` that is an `instanceOf` a `Component` defining its strain. Interactions and constraints, such as a molecule that both strains interact with, are implemented using `ComponentReference`s to link to the definitions within each cell system description. An example is shown in Figure 23.

#### 7.10.3 Cell Ratios

The proportion of cell types present in a multicellular system can be captured using `om:Measure` on the representations of cells in the design. As a best practice, the value of these measure classes is a percentage less than or equal to 100%, representing the amount of a cell type present in the system compared to all other cell types present. Therefore, the sum of all these values specified in the system will typically be equal to 100%, though this may not be the case if the system is not completely defined. An example is shown in Figure 24.

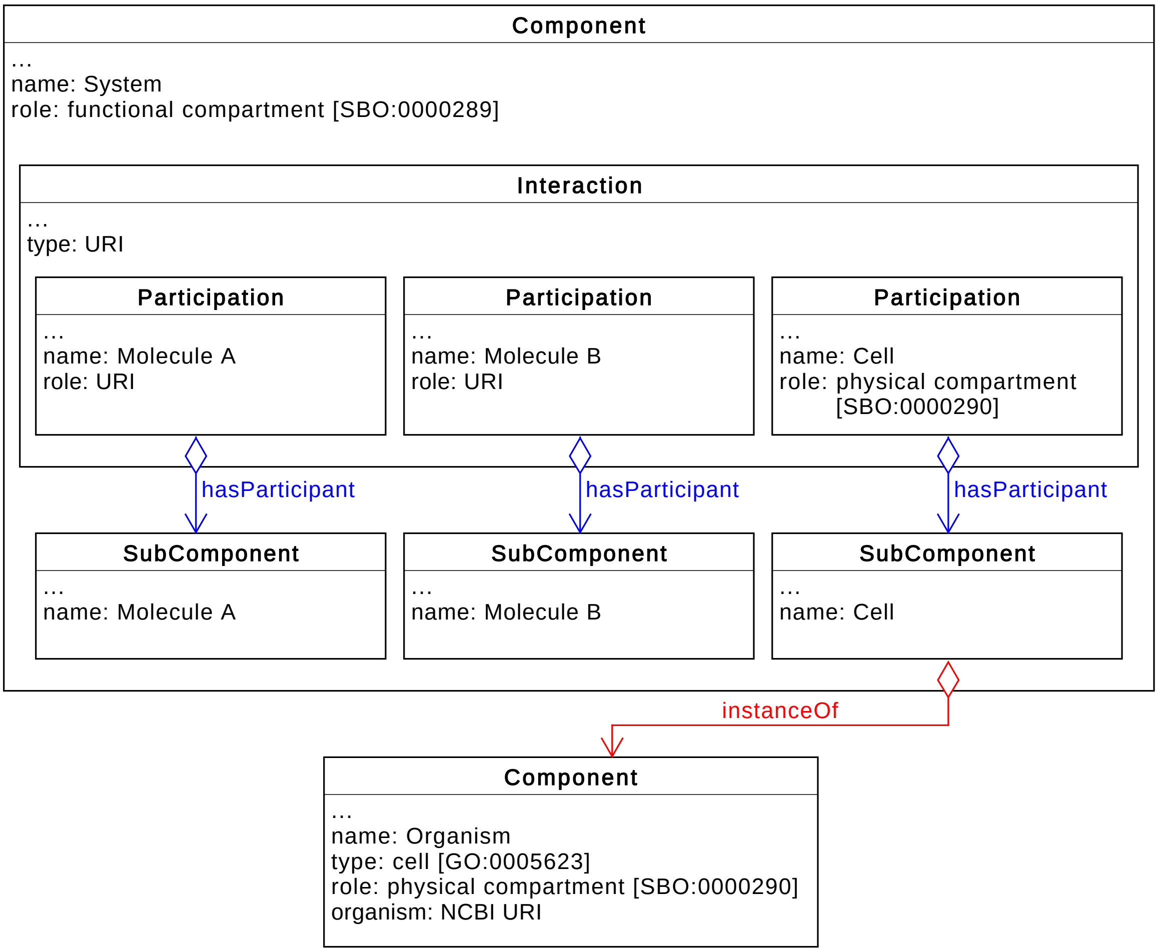

Figure 22: This is a proposed approach for capturing cell designs in SBOL. A `Component` annotated with a URI pointing to an entry in the NCBI Taxonomy Database is used to capture information about the cell's strain/species. The `Component` has a type of "Cell" from the Gene Ontology (GO), and a role of "physical compartment". Another `Component` is used to represent a system in which the cell is implemented. Entities, including the cell, are instantiated as `SubComponent`s, and processes are captured using the `Interaction` class. Processes that are contained within the cell are represented by including the cell as a participant with a role of "physical compartment".

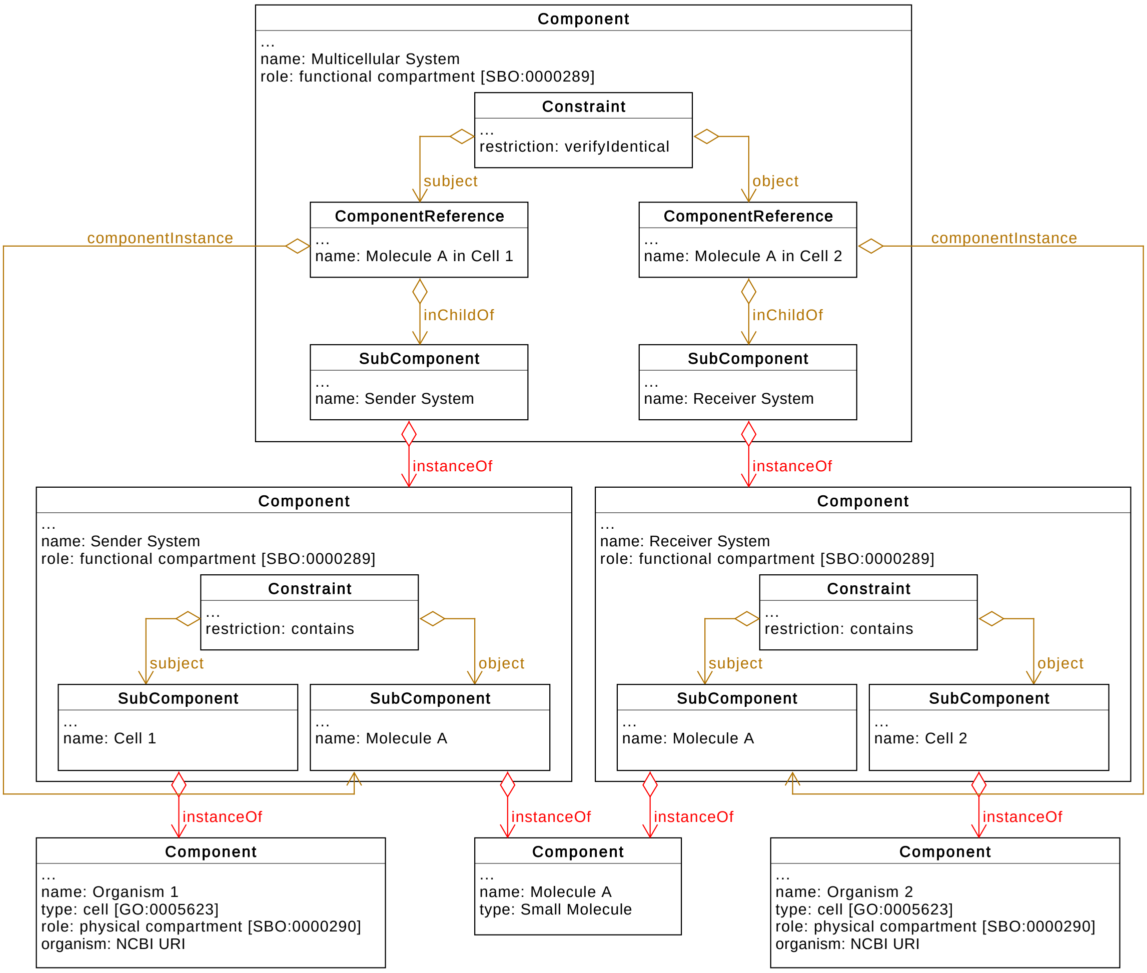

Figure 23: Captured here is a design involving two cells which both interact with the small molecule "Molecule A". Designs for the sender and receiver systems are captured using constraint to show that each of these cells interacts with the Molecule A contained within it. The overall multicellular system is represented by a `Component` with a `role` of "functional compartment", which is an SBO term. The two systems are included in this multicellular design as `SubComponent`s, and the fact that Molecule A is shared between systems is indicated with a constraint.

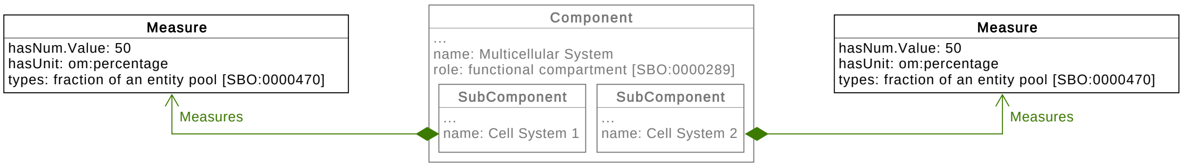

Figure 24: Annotating class instances with cellular proportions. Instances of the Measure class are used to capture the percentage of each cell type present in the multicellular system design.

## 8 SBOL RDF Serialization

In order for SBOL objects to be readily stored and exchanged, it is important that they are able to be *serialized*, i.e., converted to a sequence of bytes that can be stored in a file or exchanged over a network. The serialization format for SBOL is designed to meet several competing requirements. First, SBOL needs to support ad-hoc annotations and extensions. Second, SBOL needs to support processing by general database and semantic web software tools that have little or no knowledge of the SBOL data model. Finally, it ought to be relatively simple to write a new software implementation, so that SBOL can be readily used even in software environments where community-maintained implementations are not available.

To meet these goals, SBOL builds upon the Resource Description Framework (RDF). RDF is an abstract language for describing conceptual graph-oriented data models, and therefore does not mandate any specific serialization format. Instead, a number of different serialization formats are provided as separate specifications, such as RDF/XML, N-Triples, JSON-LD, and Turtle. These serialization formats are widely supported by RDF libraries such as rdflib for Python and Apache Jena for Java. For example, a simple SBOL definition of pLac can be serialized in RDF/XML as follows:

```
<?xml version="1.0" encoding="utf-8"?>
<rdf:RDF xmlns:rdf="http://www.w3.org/1999/02/22-rdf-syntax-ns#" xmlns:sbol="http://sbols.org/v3#">
  <sbol:Component rdf:about="http://example.com#pLac">
    <sbol:name>pLac</sbol:name>
    <sbol:description>lactose inducible promoter</sbol:description>
    <sbol:sequence rdf:resource="http://example.com#sequence"/>
  </sbol:Component>
  <sbol:Sequence rdf:about="http://example.com#sequence">
    <sbol:encoding rdf:resource="http://www.chem.qmul.ac.uk/iubmb/misc/naseq.html"/>
    <sbol:elements>caatacgcaaaccgcctctccccgcgcgttggccgattcattaatgcagctggcacga
caggtttcccgactggaaagcgggcagtgagcgcaacgcaattaatgtgagttagctcactcattaggcaccccagg
ctttacactttatgcttccggctcgtatgttgtgtggaattgtgagcggataacaatttcacaca</sbol:elements>
  </sbol:Sequence>
</rdf:RDF>
```

Alternatively, the same example can be serialized in Turtle as follows:

```
@prefix sbol: <http://sbols.org/v3#> .
@base <http://example.com#> .

<#pLac> a sbol:Component ;
    sbol:name "pLac" ;
    sbol:description "lactose inducible promoter" ;
    sbol:sequence <#sequence> .

<#sequence> a sbol:Sequence ;
    sbol:encoding <http://www.chem.qmul.ac.uk/iubmb/misc/naseq.html> ;
    sbol:elements """caatacgcaaaccgcctctccccgcgcgttggccgattcattaatgcagctggcacga
caggtttcccgactggaaagcgggcagtgagcgcaacgcaattaatgtgagttagctcactcattaggcaccccagg
ctttacactttatgcttccggctcgtatgttgtgtggaattgtgagcggataacaatttcacaca""" .
```

The SBOL namespace, which is `http://sbols.org/v3#`, is used to indicate which entities and properties in the SBOL document are defined by SBOL. For example, the URI of the type `Component` is `http://sbols.org/v3#Component`. The SBOL namespace MUST NOT be used for any entities or properties not defined in this specification. Where possible, we have re-used predicates from widely-used terminologies (such as Dublin Core DCMI Usage Board (2012)) to expose as much of the data as practical to such standard RDF tooling.

## 9 SBOL Compliance

There are different types of software compliance with respect to the SBOL specification. First, a software tool can either support all classes of the SBOL 3 data model or only its structural subset. The structural subset includes the following classes:

- `Sequence`
- `Component`
  - `SubComponent`
  - `ComponentReference`
  - `LocalSubComponent`
  - `SequenceFeature`
  - `Location`
  - `Constraint`
- `Collection`

Second, an SBOL-compliant software tool can support import of SBOL, export of SBOL, or both. If it supports both import and export, it can do so in either a lossy or lossless fashion.

In order to test import compliance, developers are encouraged to use the SBOL test files found here:
`https://github.com/SynBioDex/SBOLTestSuite`
Examples of every meaningful subset of objects are provided, including both structural-only SBOL (that is, annotated DNA sequence data) and complete tests.

In order to test export compliance, developers are encouraged to validate SBOL files generated by their software with the SBOL Validator found here:
`https://validator.sbolstandard.org`
This validator can also be used to check lossless import/export support, since it can compare the data content of files imported and exported by a software tool.

Finally, developers of SBOL-compliant tools are encouraged to notify the SBOL editors (sbol-editors@googlegroups.com) when they have determined that their tool is SBOL compliant, so their tool can be publicly categorized as such on the SBOL website.

## 10 Mapping Between SBOL 1, SBOL 2, and SBOL3

In broad strokes, the SBOL 1 standard focused on conveying physical, structural information, whereas SBOL 2 expanded the scope to include functional aspects as well. The physical information about a designed genetic construct includes the order of its constituents and their descriptions. Specifying the exact locations of these constituents and their sequences allows genetic constructs to be defined unambiguously and reused in other designs. SBOL 2 extended SBOL 1 in several ways: it extends physical descriptions to include entities beyond DNA sequences, and it added support for functional descriptions of designs. SBOL 3 refines the SBOL 2 data model to simplify the representation of common use cases.

### 10.1 Mapping between SBOL 1 and SBOL 2

Figure 25 depicts the mapping of SBOL 1.1 classes to SBOL 2.x classes, indicating corresponding classes/properties by color. The SBOL 2.x `Model` and `ModuleDefinition` classes have no SBOL 1.1 equivalent, and thus are not shown. The mapping from SBOL 1.1 to SBOL 2.x proceeds as follows:

- SBOL 1.1 `Collection` objects containing `DnaComponent` objects map to SBOL 2.x `Collection` objects that contain `CompenentDefinition` objects with DNA `type` properties.
- SBOL 1.1 `DnaComponent` objects map to SBOL 2.x `CompenentDefinition` objects with DNA `type` properties.
- SBOL 1.1 `DnaSequence` objects map to an SBOL 2.x `Sequence` objects with `IUPAC DNA encoding` properties.
- SBOL 1.1 `SequenceAnnotation` objects with `bioStart` and `bioEnd` properties map to SBOL 2.x `SequenceAnnotation` objects that contain `Range` objects.
- SBOL 1.1 `SequenceAnnotation` objects that lack `bioStart` and `bioEnd` properties map to an SBOL 2.x `SequenceFeature` objects that contain `GenericLocation` objects.
- Each SBOL 1.1 `SequenceAnnotation` also maps to an SBOL 2.x `Component`, which represents the instantiation or usage of the appropriate `CompenentDefinition`.
- Each SBOL 1.1 `precedes` property maps to an SBOL 2.x `SequenceConstraint` that specifies a `precedes restriction` property.

### 10.2 Mapping between SBOL 2 and SBOL 3

Figure 26 depicts the mapping of SBOL 2.3 classes to SBOL 3.x classes, indicating corresponding classes/properties by color. The SBOL 3.x `Namespace` class has no SBOL 2.x equivalent, and thus is not shown. The SBOL 2.x `Attachment`, `CombinatorialDerivation`, `ExperimentalData`, `Experiment`, `Implementation`, `Model`, `Participation`, `Sequence`, and `VariableComponent` classes are omitted or abstracted, since they are essentially unchanged in SBOL 3.x except for the following property renamings:

- In `Sequence`, the `encoding` property values for IUPAC map according to Table 17.
- In `VariableComponent`, the SBOL 2.x `operator` property maps to the SBOL 3.x `cardinality` property.
- In `Experiment`, the SBOL 2.x `experimentalData` property maps to the SBOL 3.x `member` property.

The mapping from SBOL 2.x to SBOL 3.x proceeds as follows:

- SBOL 2.x `ComponentDefinition` objects map to SBOL 3.x `Component` objects. The `type` property is mapped according to Table 18.

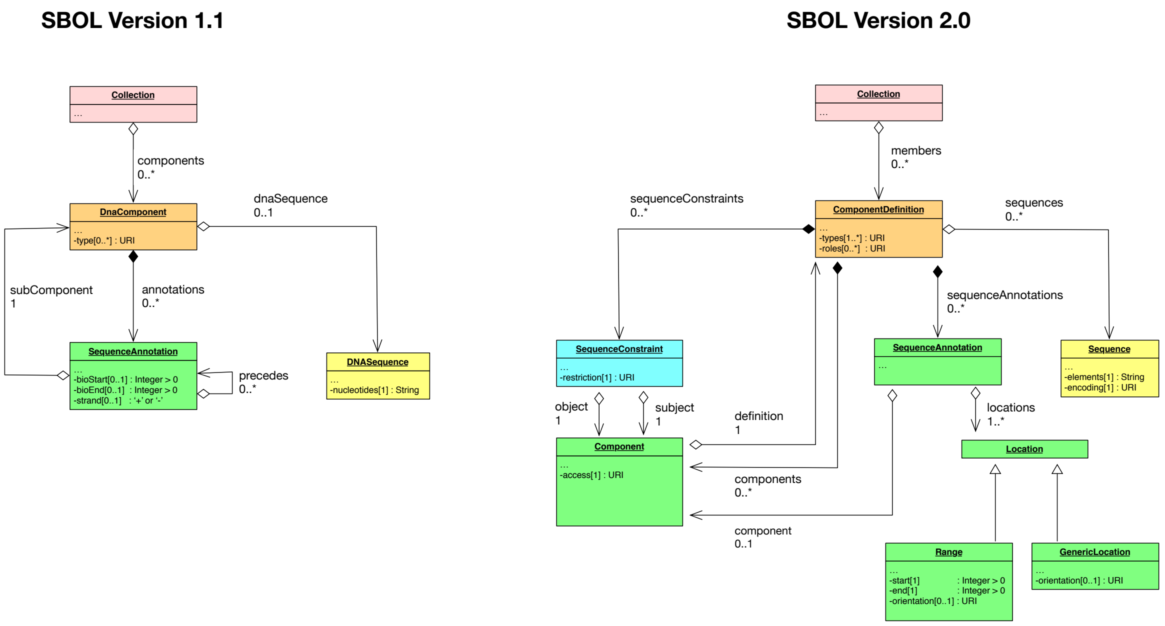

Figure 25: The mapping from the SBOL 1.1 data model to the SBOL 2.x data model, indicating corresponding classes/properties by color.

- SBOL 2.x `ModuleDefinition` objects map to SBOL 3.x `Component` objects with a `type` of `SBO:0000241` (functional entity)
- Every `FunctionalComponent` in an SBOL 2.x `ModuleDefinition` with a "direction" property that is not "none" is listed in the `Interface` of its SBOL 3.x `Component`. The mapping from direction to interface properties is: "in"–>"inputs", "out"–>"outputs", "inout" –> "nondirectional". Finally, every Component with "access"="public" and "direction"="none" is listed as "nondirectional" in the Interface.
- Every `Component` in an SBOL 2.x `ComponentDefinition` with "access"="public" is listed as "nondirectional" in the `Interface` of its SBOL 3.x `Component`.
- SBOL 2.x `Component`, `Module`, and `FunctionalComponent` objects map to SBOL 3.x `SubComponent` objects
- SBOL 2.x `SequenceAnnotation` objects map to SBOL 3.x `SequenceFeature` objects if they do not have a `component`. If they do have a `component`, their locations are added to the corresponding SBOL3 `SubComponent`.
- SBOL 2.x `SequenceConstraint` objects map to SBOL 3.x `Constraint` objects
- SBOL 2.x `MapsTo` objects are converted by transforming each `MapsTo` into two SBOL 3.x objects: a `ComponentReference` and a `Constraint`.
  - For the `ComponentReference`, the `inChildOf` attribute of this `ComponentReference` attribute references the object that has the `MapsTo` as a child, and the `hasFeature` attribute references the object referred by the `remote` attribute from the `MapsTo` object.
  - The `Constraint` links this `ComponentReference` and the `SubComponent` referred to be the `local` attribute from the `MapsTo` object. The property values of the `Constraint` depend on the value of the `refinement` value for the `MapsTo` object:
    - If the refinement is `useRemote`, then the `restriction` is `replaces`, the `subject` is the `ComponentReference` and the `object` is the `SubComponent`.
    - If the refinement is `useLocal`, then the `restriction` is `replaces`, the `subject` is the `SubComponent` and the `object` is the `ComponentReference`.

- If the refinement is verifyIdentical, then the restriction is verifyIdentical, the subject is the ComponentReference and the object is the SubComponent.
- The merge refinement was never well defined and rarely if ever used, so it has been removed from SBOL 3.x. If a merge is encountered, it SHOULD be handled as a useRemote.

- As an OPTIONAL optimization, if the SubComponent referred to by the local property of the MapsTo is a "placeholder" with no significant content apart from its MapsTo relationships, then it may be eliminated, all objects that pointed to it can point directly to the new ComponentReference instead, and all transitive constraints using it as a bridge reduced to link the endpoints directly.

| SBOL 2.x Type | SBOL 3.x Type |
|---|---|
| http://www.chem.qmul.ac.uk/iubmb/misc/naseq.html | http://sbols.org/v3#iupacNucleicAcid |
| http://www.chem.qmul.ac.uk/iupac/AminoAcid/ | http://sbols.org/v3#iupacAminoAcid |

Table 17: Mapping of Sequence encoding values for IUPAC from SBOL2 to SBOL3

| SBOL 2.x Type | SBOL 3.x Type |
|---|---|
| http://www.biopax.org/release/biopax-level3.owl#Dna | https://identifiers.org/SBO:0000251 (DNA) |
| http://www.biopax.org/release/biopax-level3.owl#DnaRegion | https://identifiers.org/SBO:0000251 (DNA) |
| http://www.biopax.org/release/biopax-level3.owl#Rna | https://identifiers.org/SBO:0000250 (RNA) |
| http://www.biopax.org/release/biopax-level3.owl#RnaRegion | https://identifiers.org/SBO:0000250 (RNA) |
| http://www.biopax.org/release/biopax-level3.owl#Protein | https://identifiers.org/SBO:0000252 (Protein) |
| http://www.biopax.org/release/biopax-level3.owl#SmallMolecule | https://identifiers.org/SBO:0000247 (Simple Chemical) |
| http://www.biopax.org/release/biopax-level3.owl#Complex | https://identifiers.org/SBO:0000253 (Non-covalent Complex) |

Table 18: Mapping of SBOL2 ComponentDefinition types to SBOL3 Component types

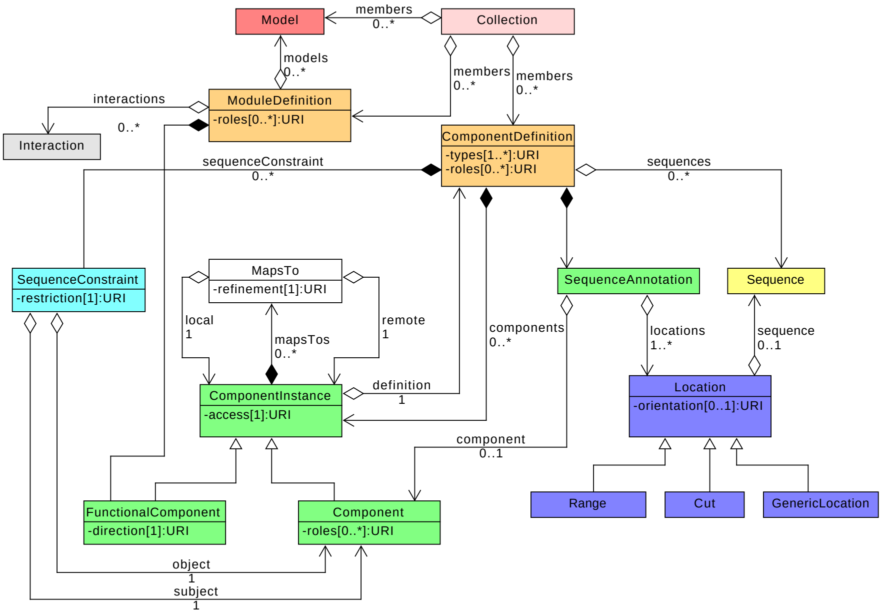

(a) SBOL 2.3

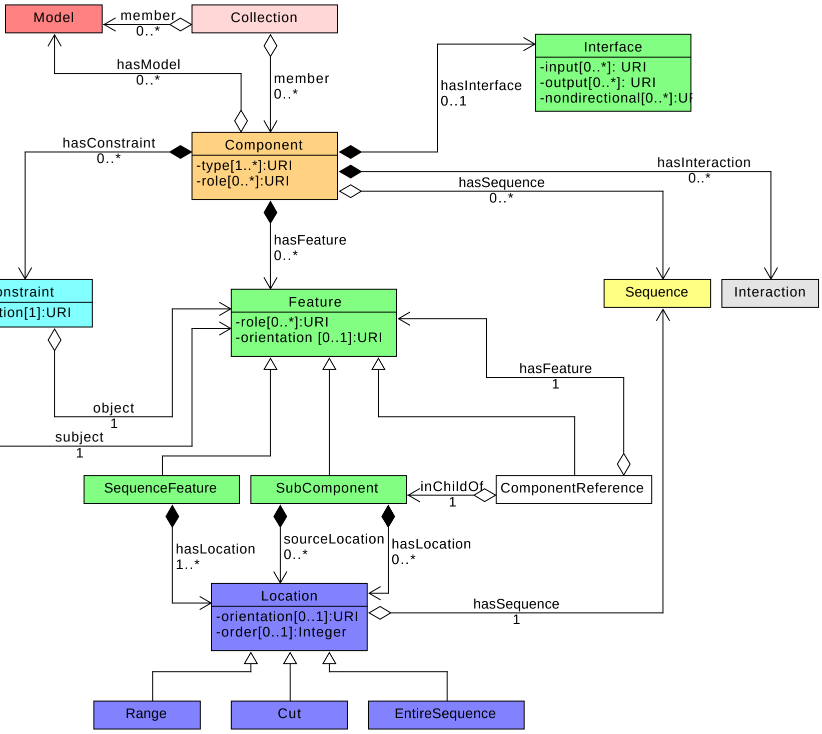

(b) SBOL 3.x

Figure 26: The mapping from the SBOL 2.3 data model to the SBOL 3.x data model, indicating corresponding classes/properties by color.

## A Complementary Standards

### A.1 Adding Provenance with PROV-O

The PROV-O ontology (https://www.w3.org/TR/prov-o/) defines a complementary data model that is leveraged by SBOL to describe provenance. Provenance is central to a range of workflow management, quality control, and attribution tasks within the Synthetic Biology design process. Tracking attribution and derivation of one resource from another is paramount for managing intellectual property purposes. Source designs are often modified in systematic ways to generate derived designs, for example, by applying codon optimization or systematically removing all of a class of restriction enzyme sites. Documenting the transformation used, and any associated parameters, makes this explicit and potentially allows the process to be reproduced systematically. If a design has been used within other designs, and is later found to be defective, it is paramount that all uses of it, including uses of edited versions of the design, can be identified, and ideally replaced with a non-defective alternative. When importing data from external sources, it is important not only to attribute the original source (for example, GenBank), but also the tool used to perform the import, as this may have made arbitrary choices as to how to represent the source knowledge as SBOL. All these activities have in common that it is necessary to track what resource, and what transformation process was applied by whom to derive an SBOL design.

This section describes a minimal subset of PROV-O terms and classes that may be used by SBOL tools to support representation of provenance[1]. Although the full-set of PROV-O terms can be used in SBOL documents, a subset of PROV-O is adopted as a best practice. It is advised that SBOL tools should at least understand this subset, defined in Figure 27. Providers of provenance information are free to make use of more of PROV-O than is described here. It is acceptable for tools that understand more than this subset to use as much as they are able. Tools that only understand this subset must treat any additional data as annotations. Tools that are not aware of SBOL provenance at all MUST maintain and provide access to this information as annotations. This specification does not state what the newly added properties must point to. As long as they are resources that are consistent with the PROV-O property domains, they are legal. For example, a `Component` may be derived from another `Component`, but it would probably not make sense for it to be derived from a `Collection`.

The most basic and general type of provenance relationship can be represented using the `prov:wasDerivedFrom` property. This relationship describes derivation of an SBOL entity from another. Any `Identified` object may be annotated with this property. More specific provenance relationships can also be defined using PROV-O, such as `prov:wasGeneratedBy`. Generation of a new object is defined by the W3C PROV-O specification as follows:

> ...the completion of production of a new entity by an activity. This entity did not exist before generation and becomes available for usage after this generation.

These relationships are leveraged in SBOL tooling for describing multi-stage synthetic biology workflows.

Synthetic biology workflows may involve multiple stages, multiple users, multiple organizations, and interdisciplinary collaborations. These workflows can be described using four core PROV-O classes: `prov:Entity`, `prov:Activity`, `prov:Agent`, and `prov:Plan`. Any SBOL `Identified` object can implicitly act as an instance of PROV-O's `prov:Entity` class. Workflow histories (retrospective provenance) and workflow specifications (prospective provenance) can be described in SBOL using `prov:Activity` objects to link `Identified` objects into workflows. An `prov:Agent` (for example a software or a person) runs an `prov:Activity` according to a `prov:Plan` to generate new entities. Resources representing `prov:Agent`, `prov:Activity` and `prov:Plan` classes should be handled as `TopLevel`, whilst `prov:Usage` and `prov:Association` resources should be treated as child `Identified` objects within their parent `prov:Activity` objects.

A design-build-test-learn SBOL ontology has been adopted for use with PROV-O classes (see Table 19). The terms *design*, *build*, *test*, and *learn* provide a high level workflow abstraction that allows tool-builders to quickly search for and isolate provenance histories relevant to their domain, while keeping track of the flow of data between different

[1] We thank Dr Paolo Missier from the School of Computing Science, Newcastle University for discussions regarding the use of PROV-O.

users working in different domains of synthetic biology. These terms SHOULD BE used on the `type` property of the `prov:Activity` class. (Note that this property is a special property added by the SBOL specification, and is not part of the original PROV-O specification.) Additionally, these terms SHOULD BE used in the `prov:hadRole` properties on `prov:Usage` to qualify how the referenced `prov:entity` is used by the parent `prov:Activity`. Logical constraints are placed on the order in which different types of `prov:Activity`s are chained into design-build-test-learn workflows. These rules additionally place constraints on the types of objects that may be used as inputs for a particular type of `prov:Activity`. For example, a *design* `prov:Usage` may be used as an input for either a *design* or *build* `prov:Activity` but MUST NOT be used as an input for a *test* `prov:Activity`. An example of how these terms are used is provided in Figure 28.

| Activity Type | URI | Description |
|---|---|---|
| Design | `http://sbols.org/v3#design` | Design describes the process by which a conceptual representation of an engineer's imagined and intended design for a biological system is created or derived. |
| Build | `http://sbols.org/v3#build` | Build describes the process by which a biological construct, sample, or clone is implemented in the laboratory. |
| Test | `http://sbols.org/v3#test` | Test describes the process of performing experimental measurements to characterize a synthetic biological construct. |
| Learn | `http://sbols.org/v3#learn` | Learn describes the process of analyzing experimental measurements to produce a new entity that represents biological knowledge. |

Table 19: Synthetic biology workflow ontology

In addition to the design-build-test-learn terms, users may also wish to include more specific terms to specify how SBOL objects are used in-house in their own recipes, protocols, or computational analyses. In fact, it is expected that the SBOL workflow ontology will be expanded over time, as users experiment with and develop their own custom ontologies. For now, however, it is RECOMMENDED that SBOL tools also include the high-level terms in Table 19 to support data exchange across interdisciplinary boundaries.

#### A.1.1 prov:Activity

A generated `prov:Entity` is linked through a `prov:wasGeneratedBy` relationship to an `prov:Activity`, which is used to describe how different `prov:Agent`s and other entities were used. An `prov:Activity` is linked through a `prov:qualifiedAssociation` to `prov:Association`s, to describe the role of agents, and is linked through `prov:qualifiedUsage` to `prov:Usage`s to describe the role of other entities used as part of the activity. Moreover, each `prov:Activity` includes optional `prov:startedAtTime` and `prov:endedAtTime` properties. When using `prov:Activity` to capture how an entity was derived, it is expected that any additional information needed will be attached as annotations. This may include software settings or textual notes. Activities can also be linked together using the `prov:wasInformedBy` relationship to provide dependency without explicitly specifying start and end times.

##### *The* `type` *property*

An `prov:Activity` MAY have one or more `type` properties, each of type `URI` that explicitly specifies the type of the provenance `prov:Activity` in more detail. If specified, it is RECOMMENDED that at least one `type` property refers to a `URI` from Table 19.

##### *The* `prov:startedAtTime` *property*

The `prov:startedAtTime` property is OPTIONAL and contains a DateTime (see Section 7.7) value, indicating when the activity started. If this property is present, then the `prov:endedAtTime` property is REQUIRED.

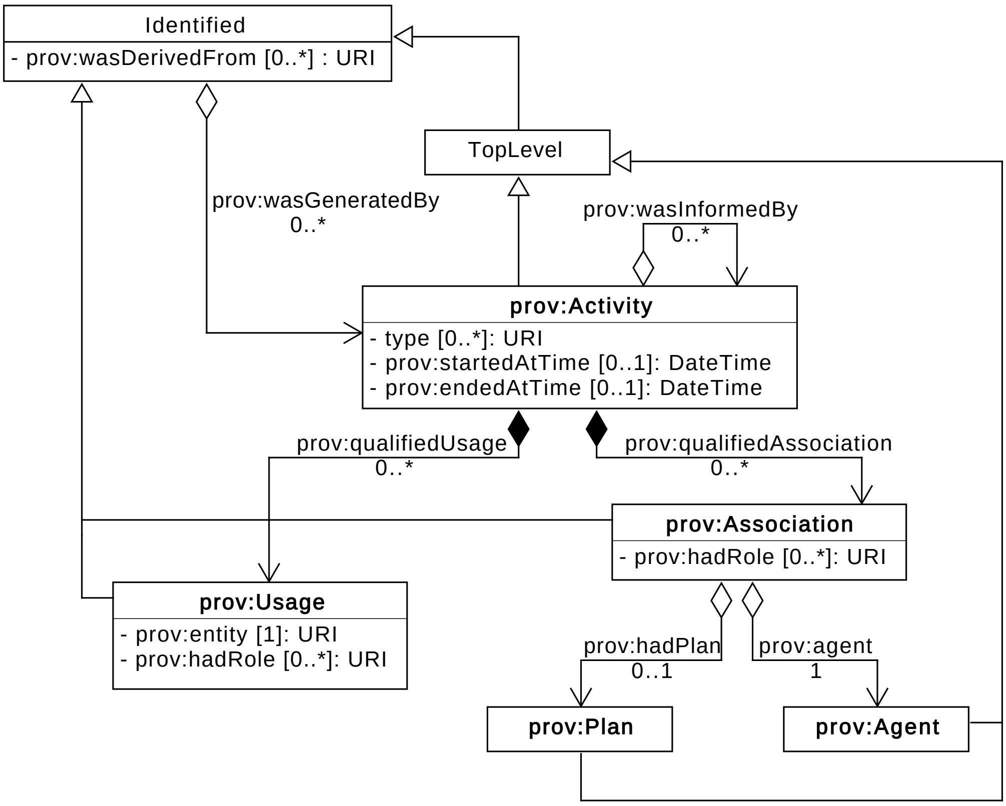

Figure 27: Relationships between SBOL and PROV-O classes. The PROV-O classes `prov:Activity`, `prov:Plan`, and `prov:Agent` all derive from `TopLevel` in the context of the SBOL data model.

##### *The* `prov:endedAtTime` *property*

The `prov:endedAtTime` property is OPTIONAL and contains a DateTime (see Section 7.7) value, indicating when the activity ended.

##### *The* `prov:qualifiedAssociation` *property*

An `prov:Activity` MAY have one or more `prov:qualifiedAssociation` properties, each of type `URI` that refers to an `prov:Association` object.

##### *The* `prov:qualifiedUsage` *property*

An `prov:Activity` MAY have one or more `prov:qualifiedUsage` properties, each of type `URI` that refers to an `prov:Usage` object.

##### *The* `prov:wasInformedBy` *property*

An `prov:Activity` MAY have one or more `prov:wasInformedBy` properties, each of type `URI` that refers to another `prov:Activity` object.

#### A.1.2 prov:Usage

How different entities are used in an prov:Activity is specified with the prov:Usage class, which is linked from an prov:Activity through the prov:Usage relationship. A prov:Usage is then linked to an prov:Entity through the prov:entity property URI and the prov:hadRole property species how the prov:Entity is used. When the prov:wasDerivedFrom property is used together with the full provenance described here, the entity pointed at by the prov:wasDerivedFrom property MUST be included in a prov:Usage.

##### The prov:entity property

The prov:entity property is REQUIRED and MUST contain a URI which MAY refer to an Identified object.

##### The prov:hadRole property

An prov:Usage MAY have one or more prov:hadRole properties, each of type URI that refers to particular term(s) describing the usage of an prov:Entity referenced by the prov:entity property. Recommended terms that are defined in Table 19 can be used to indicate how the referenced prov:Entity is being used in this prov:Activity.

#### A.1.3 prov:Association

An prov:Association is linked to an prov:Agent through the prov:agent relationship. The prov:Association includes the prov:hadRole property to qualify the role of the prov:Agent in the prov:Activity.

##### The prov:agent property

The prov:agent property is REQUIRED and MUST contain a URI that refers to an prov:Agent object.

##### The prov:hadRole property

An prov:Association MAY have one or more prov:hadRole properties, each of type URI that refers to particular term(s) that describes the role of the prov:Agent in the parent prov:Activity.

##### The prov:hadPlan property

The prov:hadPlan property is OPTIONAL and contains a URI that refers to a prov:Plan.

#### A.1.4 prov:Plan

The prov:Plan entity can be used as a place holder to describe the steps (for example scripts or lab protocols) taken when an prov:Agent is used in a particular prov:Activity.

#### A.1.5 prov:Agent

Examples of agents are a person, organization, or software tool. These agents should be annotated with additional information, such as software version, needed to be able to run the same prov:Activity again.

##### Example - Codon optimization

Codon optimization is an example of where provenance properties can be applied. The relationship between an original CDS and the codon-optimized version could simply be represented using the prov:wasDerivedFrom predicate, in a light-weight form. With more comprehensive use of the PROV ontology, the codon optimization can be represented as an prov:Activity. This prov:Activity can then include additional information, such as the prov:Agent responsible (in this case, codon-optimizing software), and additional parameters.

##### Example - Deriving strains

Bacterial strains are often derived from other strains through modifications such as gene knockouts or mutations. For example, the *Bacillus subtilis* 168 strain was derived from the NCIMB3610 strain in the 1940s through x-radiation.

*B. subtilis* 168 is a laboratory strain and has several advantages as a model organism in synthetic biology. The relationship between the original strain and the 168 strain can be represented using the `prov:wasDerivedFrom` predicate or, more comprehensively, with an `prov:Activity` describing the protocols used.

##### *Example - Design-build-test-learn Workflow*

Figure 28 illustrates one complete iteration through a design-build-test-learn cycle. The workflow begins with a `Model` which describes the hypothesized behavior of a biological device. Using a computational tool, a new Design (`Component`) is composed from biological parts, which links back to its `Model`. A genetic construct is then produced in the laboratory via an assembly protocol, and this biological sample is represented by a Build (`Implementation`). Once constructed, the Build is then characterized in the laboratory using an automated measurement protocol on a Tecan plate reader, thus generating Test data (represented by an `ExperimentalData`). Finally, a new `Model` is derived from these data using a fitting algorithm implemented in the Python programming language. The final `Model` may not match the beginning `Model`, as the observed behavior may not match the prediction.

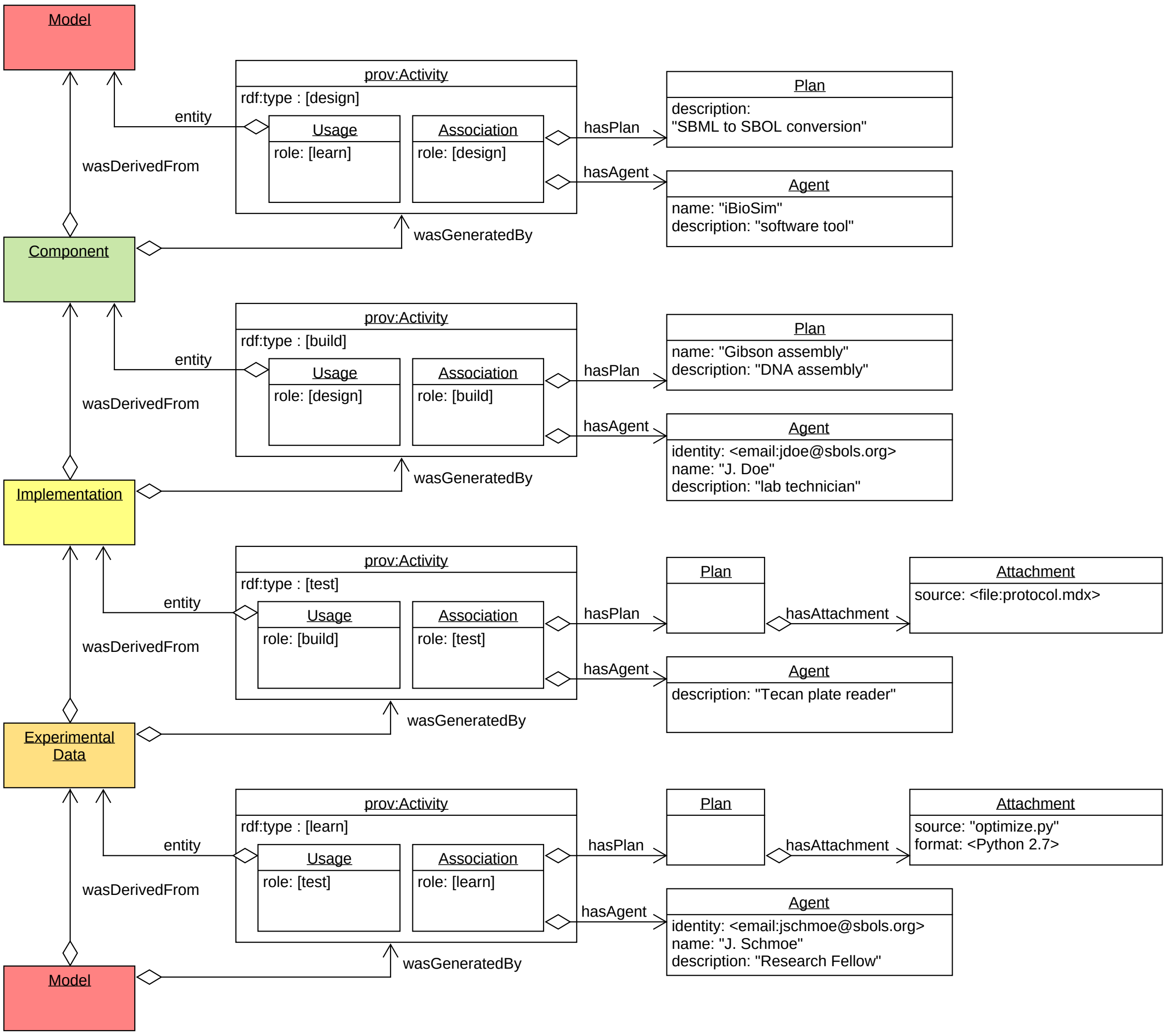

Figure 28: An example data structure representing an idealized workflow for model-based design.

***Example - Combinatorial Derivation***

As specified in the description of CombinatorialDerivation, provenance can be used to link each generated Component (or Collection thereof) back to the source form which it was derived. In particular, each derived design links with prov:wasDerivedFrom to the CombinatorialDerivation that it was derived from. Also, each SubComponent has a prov:wasDerivedFrom linking it to the SubComponent within the template that it is derived from. The advantage of these provenance links is that they provide sufficient information to validate that this derived design has been properly derived from the specified CombinatorialDerivations.

### A.2 Adding Measures/Parameters with OM

There are at least two well-established cases for including measures/parameters and their associated units in SBOL design specifications. These use cases are the specification of genetic circuit designs and their associated parameters (such as rates of transcription) and the specification of environmental conditions for biological system designs (such as growth media concentrations and temperatures). In the first use case, parameters are necessary to enable the generation of quantitive models of circuit behavior from circuit design specifications. In the second use case, measures are necessary to define experimental conditions and enable the analysis of system behavior or characterization with respect to environmental context.

The Ontology of Units of Measure (OM) (http://www.ontology-of-units-of-measure.org/resource/om-2) already defines a data model for representing measures and their associated units. Here, a subset of OM is adopted by SBOL to describe these concepts for biological design specifications. As shown in Figure 29, SBOL leverages three of the base classes defined by the OM: om:Measure, om:Unit and om:Prefix. A om:Measure links a numerical value to a om:Unit, which may or may not have a om:Prefix (e.g. centi, milli, micro, etc.). As these classes are adopted by SBOL, om:Measure is treated as a subclass of Identified, while om:Unit and om:Prefix are treated as subclasses of TopLevel. In addition, SBOL adopts the following OM om:Unit subclasses: om:SingularUnit, om:CompoundUnit, om:UnitMultiplication, om:UnitDivision, om:UnitExponentiation, and om:PrefixedUnit. Lastly, SBOL adopts the following om:Prefix subclasses from OM: om:SIPrefix and om:BinaryPrefix.

SBOL-compliant tools are allowed to read, write, and modify data belonging to OM classes other than those described here, but this specification does not provide any guidance for the interpretation or use of these data in the context of SBOL.

#### A.2.1 om:Measure

The purpose of the om:Measure class is to link a numerical value to a om:Unit.

***The om:hasNumericalValue property***

The om:hasNumericalValue property is REQUIRED and MUST contain a single xsd:float.

***The om:hasUnit property***

The om:hasUnit property is REQUIRED and MUST contain a URI that refers to a om:Unit. The OM provides URIs for many existing instances of the om:Unit class for reference (for example, http://www.ontology-of-units-of-measure.org/resource/om-2/gramPerLitre).

***The type property***

A om:Measure MAY have one or more type properties, each is of type URI. It is RECOMMENDED that one of these URIs identify a term from the Systems Description Parameter branch of the Systems Biology Ontology (SBO) (http://www.ebi.ac.uk/sbo/main/). This type property of the om:Measure class is not specified in the OM and is added by SBOL to describe different types of parameters (for example, rate of reaction is identified by the SBO term http://identifiers.org/biomodels.sbo/SBO:0000612).

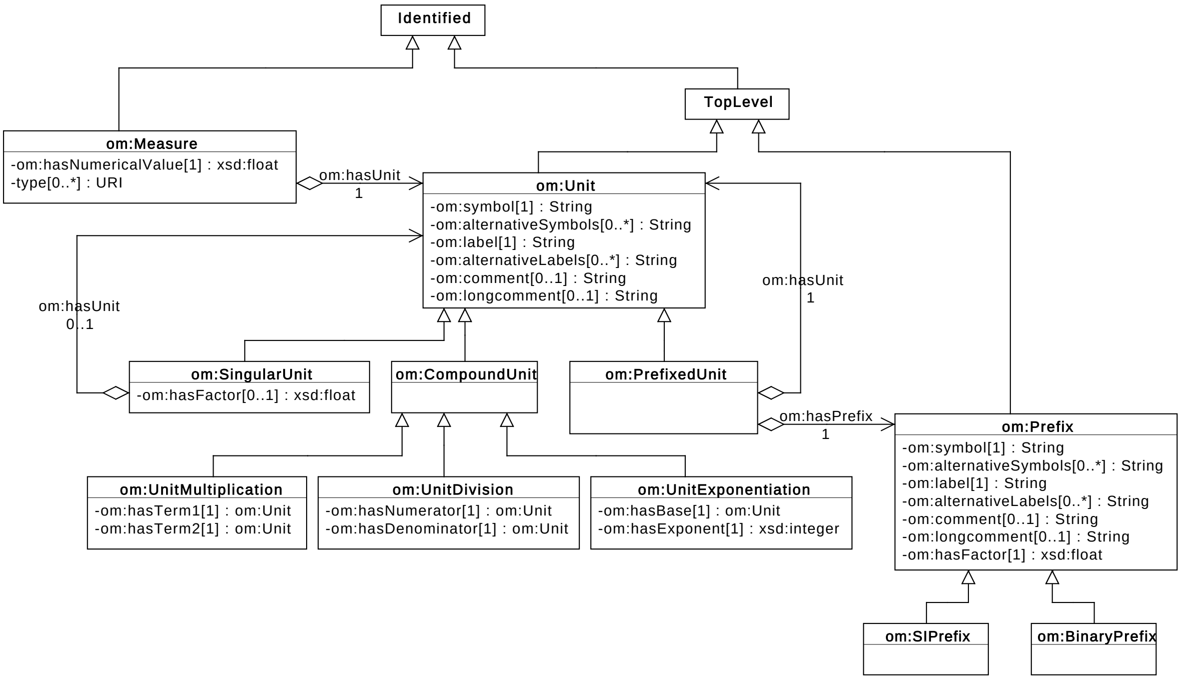

Figure 29: OM classes adopted by SBOL and their subclass relationships to `Identified` and `TopLevel`

#### A.2.2 om:Unit

As adopted by SBOL, `om:Unit` is an abstract class that is extended by other classes to describe units of measure using a shared set of properties.

##### *The `om:symbol` property*

The `om:symbol` property is REQUIRED and MUST contain a `String`. This `String` is commonly used to abbreviate the unit of measure's name. For example, the unit of measure named "gram per liter" is commonly abbreviated using the `String` "g/l".

##### *The `om:alternativeSymbols` property*

The `om:alternativeSymbols` property is OPTIONAL and MAY contain a set of `String`s. This property can be used to specify alternative abbreviations other than that specified using the `om:symbol` property.

##### *The `om:label` property*

The `om:label` property is REQUIRED and MUST contain a `String`. This `String` is a common name for the unit of measure and SHOULD be identical to any `String` contained by the `name` property inherited from `Identified`.

##### *The `om:alternativeLabels` property*

The `om:alternativeLabels` property is OPTIONAL and MAY contain a set of `String`s. This property can be used to specify alternative common names other than that specified using the `om:label` property.

##### *The `om:comment` property*

The `om:comment` property is OPTIONAL and MAY contain a `String`. This `String` is a description of the unit of measure and SHOULD be identical to any `String` contained by the `description` property inherited from `Identified`.

##### *The `om:longcomment` property*

The om:longcomment property is OPTIONAL and MAY contain a String. This String is a long description of the unit of measure and SHOULD be longer than any String contained by the om:comment property.

#### A.2.3 om:SingularUnit

The purpose of the om:SingularUnit class is to describe a unit of measure that is not explicitly represented as a combination of multiple units, but could be equivalent to such a representation. For example, a joule is considered to be a om:SingularUnit, but it is equivalent to the multiplication of a newton and a meter.

##### *The `om:hasUnit` property*

The om:hasUnit is OPTIONAL and MAY contain a URI. This URI MUST refer to another om:Unit. The om:hasUnit propery can be used in conjunction with the om:hasFactor property to specify whether a om:SingularUnit is equivalent to another om:Unit multiplied by a factor. For example, an angstrom is equivalent to $10^{-10}$ meters.

##### *The `om:hasFactor` property*

The om:hasFactor property is OPTIONAL and MAY contain a xsd:float. If the om:hasFactor property of a om:SingularUnit is non-empty, then its om:hasUnit property SHOULD also be non-empty.

#### A.2.4 om:CompoundUnit

As adopted by SBOL, om:CompoundUnit is an abstract class that is extended by other classes to describe units of measure that can be represented as combinations of multiple other units of measure.

#### A.2.5 om:UnitMultiplication

The purpose of the om:UnitMultiplication class is to describe a unit of measure that is the multiplication of two other units of measure.

##### *The `om:hasTerm1` property*

The om:hasTerm1 property is REQUIRED and MUST contain a URI that refers to another om:Unit. This om:Unit is the first multiplication term.

##### *The `om:hasTerm2` property*

The om:hasTerm2 property is REQUIRED and MUST contain a URI that refers to another om:Unit. This om:Unit is the second multiplication term. It is okay if the om:Unit referred to by om:hasTerm1 is the same as that referred to by om:hasTerm2.

#### A.2.6 om:UnitDivision

The purpose of the om:UnitDivision class is to describe a unit of measure that is the division of one unit of measure by another.

##### *The `om:hasNumerator` property*

The om:hasNumerator property is REQUIRED and MUST contain a URI that refers to another om:Unit.

##### *The `om:hasDenominator` property*

The om:hasDenominator property is REQUIRED and MUST contain a URI that refers to another om:Unit.

#### A.2.7 om:UnitExponentiation

The purpose of the `om:UnitExponentiation` class is to describe a unit of measure that is raised to an integer power.

##### *The `om:hasBase` property*

The `om:hasBase` property is REQUIRED and MUST contain a `URI` that refers to another `om:Unit`.

##### *The `om:hasExponent` property*

The `om:hasExponent` property is REQUIRED and MUST contain an `xsd:integer`.

#### A.2.8 om:PrefixedUnit

The purpose of the `om:PrefixedUnit` class is to describe a unit of measure that is the multiplication of another unit of measure and a factor represented by a standard prefix such as "milli," "centi," "kilo," etc.

##### *The `om:hasUnit` property*

The `om:hasUnit` property is REQUIRED and MUST contain a `URI` that refers to another `om:Unit`.

##### *The `om:hasPrefix` property*

The `om:hasPrefix` property is REQUIRED and MUST contain a `URI` that refers to a `om:Prefix`.

#### A.2.9 om:Prefix

As adopted by SBOL, `om:Prefix` is an abstract class that is extended by other classes to describe factors that are commonly represented by standard unit prefixes. For example, the factor $10^{-3}$ is represented by the standard unit prefix "milli."

##### *The `om:symbol` property*

The `om:symbol` property is REQUIRED and MUST contain a `String`. This `String` is commonly used to abbreviate the name of the unit prefix. For example, the `String` "m" is commonly used to abbreviate the name "milli."

##### *The `om:alternativeSymbols` property*

The `om:alternativeSymbols` property is OPTIONAL and MAY contain a set of `String`s. This property can be used to specify alternative abbreviations other than that specified using the `om:symbol` property.

##### *The `om:label` property*

The `om:label` property is REQUIRED and MUST contain a `String`. This `String` is a common name for the unit prefix and SHOULD be identical to any `String` contained by the `name` property inherited from `Identified`.

##### *The `om:alternativeLabels` property*

The `om:alternativeLabels` property is OPTIONAL and MAY contain a set of `String`s. This property can be used to specify alternative common names other than that specified using the `om:label` property.

##### *The `om:comment` property*

The `om:comment` property is OPTIONAL and MAY contain a `String`. This `String` is a description of the unit prefix and SHOULD be identical to any `String` contained by the `description` property inherited from `Identified`.

##### *The `om:longcomment` property*

The `om:longcomment` property is OPTIONAL and MAY contain a `String`. This `String` is a long description of the unit of measure and SHOULD be longer than any `String` contained by the `om:comment` property.

##### *The `om:hasFactor` property*

The `om:hasFactor` property is REQUIRED and MUST contain an xsd:float.

#### *A.2.10 om:SIPrefix*

The purpose of the `om:SIPrefix` class is to describe standard SI prefixes such as “milli,” “centi,” “kilo,” etc.

#### *A.2.11 om:BinaryPrefix*

The purpose of the `om:BinaryPrefix` class is to describe standard binary prefixes such as “kibi,” “mebi,” “gibi,” etc. These prefixes commonly precede units of information such as “bit” and “byte.”

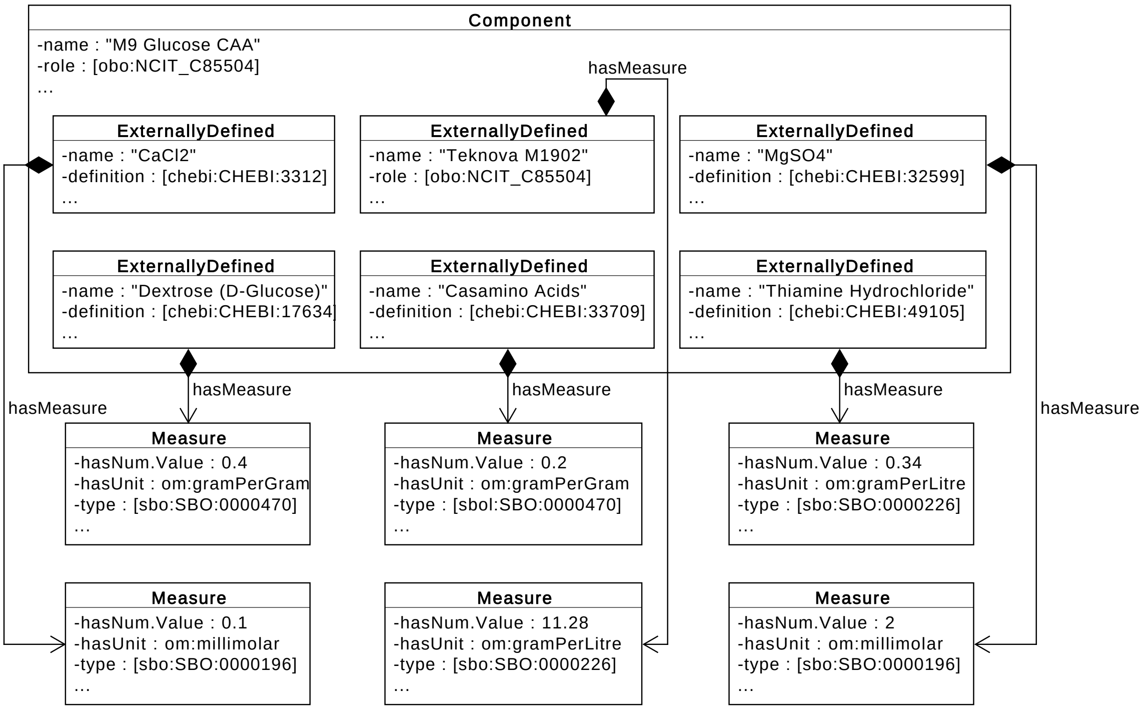

Figure 30: Growth media recipe represented using instances of the `om:Measure` and `om:Unit` classes from the OM.
